# In Vitro and in Vivo Imaging of Nitroxyl with Copper Fluorescent Probe in Living Cells and Zebrafish

**DOI:** 10.3390/molecules23102551

**Published:** 2018-10-06

**Authors:** Sathyadevi Palanisamy, Yu-Liang Wang, Yu-Jen Chen, Chiao-Yun Chen, Fu-Te Tsai, Wen-Feng Liaw, Yun-Ming Wang

**Affiliations:** 1 Department of Biological Science and Technology, Institute of Molecular Medicine and Bioengineering, Center For Intelligent Drug Systems and Smart Bio-devices (IDS2B), National Chiao Tung University, 75 Bo-Ai Street, Hsinchu 300, Taiwan; sathyadevi85@gmail.com (S.P.); littlestars2@livemail.tw (Y.-L.W.); c2h5oh.eric@gmail.com (Y.-J.C.); 2 Department of Radiology, Faculty of Medicine, College of Medicine, Kaohsiung Medical University, Kaohsiung 807, Taiwan; ccy0103@hotmail.com; 3 Department of Medical Imaging, Kaohsiung Medical University Hospital, Kaohsiung 807, Taiwan; 4 Department of Chemistry, National Tsing Hua University, Hsinchu 30043, Taiwan; fttsai@mx.nthu.edu.tw (F.-T.T.); wfliaw@mx.nthu.edu.tw (W.-F.L.); 5 Department of Biomedical Science and Environmental Biology, Kaohsiung Medical University, Kaohsiung 807, Taiwan

**Keywords:** nitroxyl, fluorescent probe, living system, zebrafish (*Danio rerio*)

## Abstract

Nitroxyl (HNO) plays a critical role in many physiological processes which includes vasorelaxation in heart failure, neuroregulation, and myocardial contractility. Powerful imaging tools are required to obtain information for understanding the mechanisms involved in these in vivo processes. In order to develop a rapid and high sensitive probe for HNO detection in living cells and the zebrafish model organism, 2-((2-(benzothiazole-2yl)benzylidene) amino)benzoic acid (AbTCA) as a ligand, and its corresponding copper(II) complex Cu(II)-AbTCA were synthesized. The reaction results of Cu(II)-AbTCA with Angeli’s salt showed that Cu(II)-AbTCA could detect HNO quantitatively in a range of 40–360 µM with a detection limit of 9.05 µM. Furthermore, Cu(II)-AbTCA is more selective towards HNO over other biological species including thiols, reactive nitrogen, and reactive oxygen species. Importantly, Cu(II)-AbTCA was successfully applied to detect HNO in living cells and zebrafish. The collective data reveals that Cu(II)-AbTCA could be used as a potential probe for HNO detection in living systems.

## 1. Introduction

Nitrogen oxides are considered as essential elements of the biochemistry and physiology of living organisms. Biological actions accompanied with nitrogen oxides are the topic of major and current research attention. Nitroxyl (HNO), is a simple triatomic species related to nitric oxide (NO), a one-electron reduced congener but HNO possesses unique chemical and biological characteristics that differentiate it from NO [1,2]. HNO is a highly reactive electrophile, and reports on HNO chemistry includes various examples of reactivity with oxidants, nucleophiles, and metalloproteins [3].

HNO is considered a vasodilator and positive inotropic agent which can be used for effective heart failure treatment [4]. Angeli’s salt trigger vasorelaxation in isolated large channels, [5,6] HNO also targets K^+^ channels to regulate vascular functions. Reports showed that both K_v_ and K_ATP_ channels were affected during HNO mediated vascular and non-vascular smooth muscle relaxations [7,8]. The vasodilation ability of HNO donors and their potential to target signaling pathways are distinctive from that of NO and offers beneficial effects over traditional nitro vasodilators. Nitroxyl also interacts with thiol residues on the N-methyl-D-aspartate receptor which plays multidisciplinary functions in the central nervous system [9]. In addition, HNO aggravates ischemia-reperfusion injury when applied during reperfusion whereas NO exhibits quite opposite effects [10].

In recent years, various detection techniques were developed for HNO detection, which include HNO trapping methods using metalloporphyrins [11], thiols [12], and phosphines [13]. Even submicromolar HNO concentrations in aqueous media have been detected using inlet mass spectrometry [14]. Many other techniques are also available for HNO detection such as colorimetry, electrochemistry, chemiluminescence, and electron paramagnetic resonance [15,16,17]. However, these techniques are not suitable to visualize HNO in vitro and in vivo. These days, a powerful and propitious fluorescence imaging method has been successfully established to examine bioactive species in living systems. Recently, a variety of highly fluorescent probes suitable for real time imaging were developed and facilitated an advancement in cell biology and therapeutics imaging [18,19,20]. Some studies have demonstrated that HNO can react with triphenylphosphine to form corresponding phosphine oxide and azaylide. This azaylide can undergo intramolecular ester aminolysis to generate alcohol and amide [13,21]. This type of reaction can provide a promising opportunity for HNO sensing chemically. Reports are available describing the design of two-photon fluorescent probes, which are suitable for monitoring HNO not only in living cells, but also in living tissues [22]. Another previously reported fluorescent probe for HNO was based on nitroxide to hydroxylamine conversion by HNO [23]. Another perspective for HNO sensing relies on HNO-induced Cu(II) to Cu(I) reduction. The BODIPY-based CuBOT1 complex [24,25], coumarin-based derivatives (CuCOT1) [26], and benzoresorufin-based CuBRNO series [27] are the probes available in the literature that follow a Cu(II) to Cu(I) reduction strategy. A near-infrared probe following the Cu(II) to Cu(I) reduction strategy was developed, but the probe is not sensitive enough and the fluorescence increment is only about 5-fold [28]. HNO reacts with oxidized metals and forms reductive nitrosylation products: Fe(III) + HNO→Fe(II)NO + H^+^ [3,29].

Regardless of the benefits in the establishment of HNO fluorescent probes, these reports indicate that the majority of the sensors are susceptible to be interfered with in bio-imaging applications by various biological reductant species such as hydrogen sulfide, glutathione, and ascorbic acid in living matrixes [25,26,27,30,31]. On the contrary HNO and NO are interconvertible using superoxide dismutase [14,32]. Recently, nitroaromatic explosives were detected using conjugated polymers based on fluorescence quenching by the amplification of the fluorescent signal with high sensitivity [33,34,35]. This kind of methodology has been demonstrated to be promising due to its advantages of high sensitivity, rapidity, non-destructive methodology and direct visual interpretation for short wavelength fluorescent probes [36]. Also, not many turn-off fluorescent copper probes are available for HNO detection in the literature to operate under a fluorescent quenching mechanism. High selectivity and sensitivity are also required for HNO detection by fluorescent probes. Hence, the fabrication of fluorescent probes with high sensitivity, selectivity, and the reductant-resistant ability for HNO detection became our target.

In this work, we set out to design a copper(II) complex, Cu(II)-AbTCA, as a sensor for nitroxyl detection by incorporating the imine-functionalized thiazole unit AbTCA with Cu^2+^. It is known from previous reports that copper-containing fluorescent probes readily react with nitroxyl and further undergo reduction processes [26,37]. Therefore, we expected that the prepared copper complex, Cu(II)-AbTCA probe titrating against Angeli’s salt would reduce from a Cu^2+^ to a Cu^+^ state and alter the fluorescence of the probe that could then be analyzed using fluorescence methods. UV-visible, fluorescence, selectivity, time-dependent, and pH studies were conducted to illustrate the fluorescence behavior of Cu(II)-AbTCA towards HNO detection. Indeed, this copper complex sensor, Cu(II)-AbTCA featured a “turn-off” fluorescence response for HNO with excellent selectivity over NO and other biological species in aqueous environments. Detection of nitroxyl released from dinitrosyliron complex (DNIC), [PPN][Fe(NO)_4_], and thiophenol were examined using the Cu(II)-AbTCA probe. In addition, biological studies involving cytotoxicity, HNO detection in living cells. In vivo imaging of HNO in the model organism zebrafish were also investigated.

## 2. Results

### 2.1. Design Strategy, Synthesis, and Properties of AbTCA and Cu(II)-AbTCA

The synthetic route provided in Scheme S1 (see Supplementary Materials) was used to synthesize the compounds AbTCA and Cu(II)-AbTCA in high yields under relatively mild conditions. In brief, the imine functionalized thiazole unit, AbTCA was synthesized in a high yield of 68% by the condensation reaction between 2-(2-aminophenyl)benzothiazole and 2-carboxbenzaldehyde. Subsequently, treatment of AbTCA with CuCl_2_ in ethanol afforded Cu(II)-AbTCA with a yield of about 27% and high purity. The formation of AbTCA was confirmed using NMR, HPLC, and mass spectral methods (Figures S1–S5). The Cu(II)-AbTCA complex formation was confirmed by HRESI-MS spectral studies (Figure S6). The fluorescence quantum yield values of AbTCA and Cu(II)-AbTCA were found to be 0.15 and 0.51, respectively.

Later, the optical properties of Cu(II)-AbTCA were examined in the presence or absence of Angeli’s salt upon conducting different experiments such as UV-visible, fluorescence, selectivity, time-dependent, and studies examining the effect of pH. Dinitrosyliron complex (DNIC), [PPN][Fe(NO)_4_], and thiophenol were employed to generate nitroxyl and the subsequent detection of nitroxyl release was demonstrated by utilizing Cu(II)-AbTCA probe via an in situ method with the help of fluorescence analysis. Furthermore, we evaluated the biological properties of the Cu(II)-AbTCA probe, including cytotoxicity and cellular imaging (both in vitro and in vivo) towards HNO. A literature report states that the complete balance between lipophilicity and hydrophilicity makes chemodosimeters suitable for cell permeability and intracellular fluorescence imaging [38]. Hence, a small amount of DMSO was added as co-solvent in this study to perform HNO sensing using Cu(II)-AbTCA in PBS (10 mM, pH 7.4 containing 1% DMSO and 0.01 M CTAB) in all the experiments.

### 2.2. Assessment of UV-Visible and Fluorescence Response to HNO

**Figure 1:**
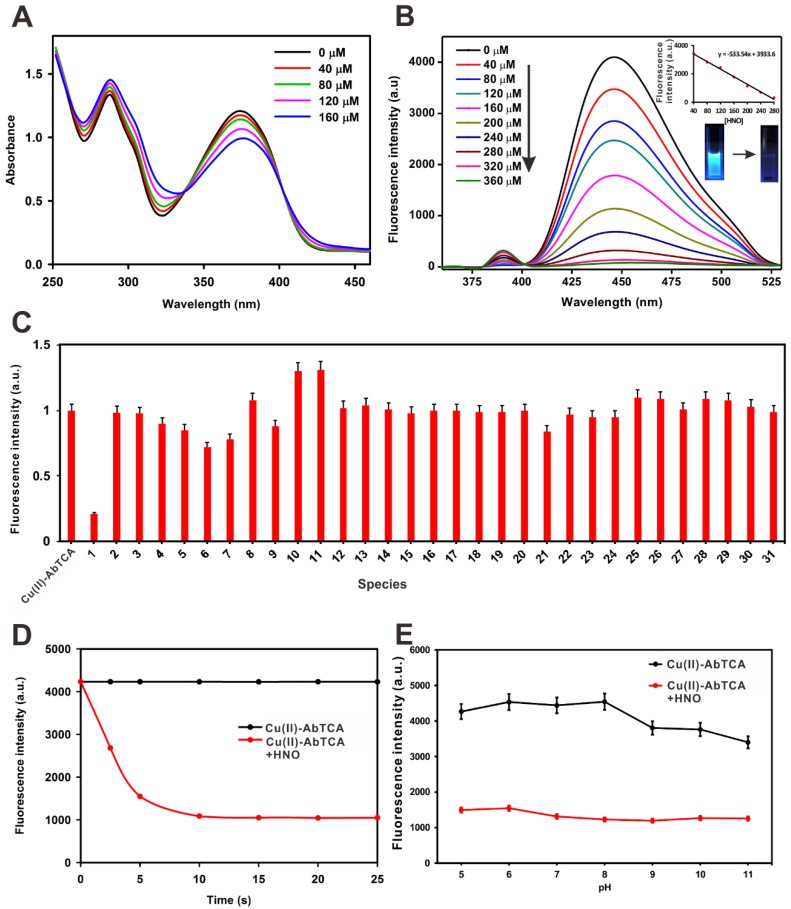
(**A**) UV-visible spectra of Cu(II)-AbTCA (40 μM) with the addition of increasing Angeli’s salt (40–160 μM) in PBS (10 mM, pH 7.4 containing 1% DMSO and 0.01 M CTAB); (**B**) Fluorescence spectra of Cu(II)-AbTCA (40 μM) with the addition of increasing concentrations of Angeli’s salt (40–360 μM) in PBS (10 mM, pH 7.4 containing 1% DMSO and 0.01 M CTAB). Each spectrum was recorded after 5 min. Inset: the relationship between maximum fluorescence intensity and Angeli’s salt concentration; (C) Fluorescence responses of Cu(II)-AbTCA to various analytes in PBS (10 mM, pH 7.4 containing 1% DMSO and 0.01 M CTAB). Bars represent the relative fluorescent intensity of Cu(II)-AbTCA (40 μM) with Angeli’s salt (200 μM) (1); analytes (1 mM) NO^+^ (2); NO (3); Cys (4); FeCl_3_ (5); NaClO_4_ (6); NaNO_3_ (7); Hys (8); NaHS (9); NaNO_2_ (10); H_2_O_2_ (11); ONOO^−^ (12); Thr (13); Leu (14); Pro (15); Ala (16); Asn (17); Phe (18); Arg (19); Gln (20); Ser (21); Ile (22); Lys (23); Trp (24); His (25); Val (26); NaI (27); NaBr (28); NaCl (29); Glu (30); and RSNO (31) over the original emission of free Cu(II)-AbTCA; (**D**) Time dependent fluorescence spectra of Cu(II)-AbTCA (40 μM) with Angeli’s salt (200 μM) in PBS (10 mM, pH 7.4 containing 1% DMSO and 0.01 M CTAB) for 25 s; (**E**) pH effect of Cu(II)-AbTCA (40 μM) with the addition of Angeli’s salt concentration (200 μM) in PBS (10 mM, pH 7.4 containing 1% DMSO and 0.01 M CTAB).

For typical optical measurements, Cu(II)-AbTCA was diluted to 40 μM by adding an appropriate amount of stock solution and then diluting it for 2 mL using PBS (10 mM, pH 7.4 containing 1% DMSO and 0.01 M CTAB). It was observed that the addition of [Cu-(AbTCA)] in 9:1 (*v*/*v*) water (H_2_O): dimethyl sulfoxide (DMSO) leads to rapid precipitation of the complex. In order to avoid the precipitate formation and to study the reaction of HNO, cetyltrimethylammonium bromide (CTAB) (0.1 M) was used as an additive [39,40]. UV-visible and fluorescence spectra upon HNO addition were measured. Firstly, UV-visible spectra of Cu(II)-AbTCA were measured in the absence and presence of Angeli’s salt (Figure 1a). Free Cu(II)-AbTCA (40 µM) displayed two well distinct absorption bands one centered at 284 nm and another positioned at 375 nm. When the concentration of Angeli’s salt is increased gradually from 40–160 µM, an increment in the absorbance positioned at 375 nm with a simultaneous decrease in the absorbance at 284 nm was observed. In addition, two isosbestic points were also developed at 338 and 404 nm. Free Cu(II)-AbTCA (40 μM) shows two emission bands centered at 390 and 450 nm (λ_ex_ = 375 nm; Figure 1b). When titrated with different concentrations of Angeli’s salt ranging from 40–360 µM, a ratiometric fluorescence response was observed. Fluorescence titration of a Cu(II)-AbTCA solution with Angeli’s salt demonstrated that its emission band at 390 nm increased very slightly, while the band at 450 nm underwent a noticeable reduction simultaneously (Figure 1b). The distinct gap between the two bands is too small, which makes this probe unfavorable for the dual emission ratiometric imaging. Finally, the small increment of the emission band at 390 nm was ignored and the major reduction in the 450 nm emission band was considered to carry out further studies. The fluorescence of Cu(II)-AbTCA was completely turned off by the addition of Angeli’s salt as the HNO interaction with metal ion causes reduction of Cu(II) to diamagnetic Cu(I) ion. The fluorescence quenching was observed by the naked eye (Figure 1b). 

In addition, an excellent linearity was obtained between fluorescence intensity and Angeli’s salt concentration with a detection limit of 9.05 μM which is higher than that of the reported literature [34]. Together, these experimental results clearly demonstrated that Cu(II)-AbTCA has potential to detect HNO quantitatively using a fluorescence spectroscopic method with high sensitivity.

### 2.3. Selectivity Studies

To test whether the specificity of Cu(II)-AbTCA for HNO is high enough to monitor nitroxyl even in the presence of other biological molecules, the Cu(II)-AbTCA (40 μM) responses towards Angeli’s salt (200 µM) were measured in the presence of 1 mM concentrations of biologically relevant reactive oxygen, nitrogen, and sulphur species, cations/anions, and free radicals. Results showed that nearly no changes in the fluorescence intensity of Cu(II)-AbTCA were observed in the presence of various biologically relevant species (Figure 1c). These results indicated that Cu(II)-AbTCA has high selectivity towards HNO in the presence of different biological species. The stability and the interference of the complex was checked with other metal ions (Figure S7).

### 2.4. Reaction Time Effects on HNO Sensing 

Response time is an essential factor for chemosensors and hence the time needed for the reaction to occur between Cu(II)-AbTCA (40 μM) with Angeli’s salt (200 μM) was investigated. During the reaction between Cu(II)-AbTCA and Angeli’s salt, the fluorescence intensity of Cu(II)-AbTCA is decreased with response time and the levels almost turned off when the response time is greater than 10 s (Figure 1d). Therefore, it can be strongly said that Cu(II)-AbTCA serves as a rapid sensor in HNO detection under physiological conditions.

### 2.5. Effects of pH Studies

For a fluorescence probe Cu(II)-AbTCA, the effects of sensing performance with various pH levels were evaluated and hence, the titration was performed to examine the pH effect on fluorescence of Cu(II)-AbTCA (40 µM) upon treatment with Angeli’s salt (200 μM). Emission changes of Cu(II)-AbTCA probe (40 μM) alone were also measured. Results indicated that Cu(II)-AbTCA produced strong fluorescence intensity whereas Angeli’s salt-added Cu(II)-AbTCA probe showed a minimal effect in the pH range between 5.0–11.0 (Figure 1e). These results indicate that Cu(II)-AbTCA can be expected to work well under the mentioned physiological conditions.

### 2.6. Mechanism of Cu(II)-Abtca in Sensing HNO

**Scheme 1:**
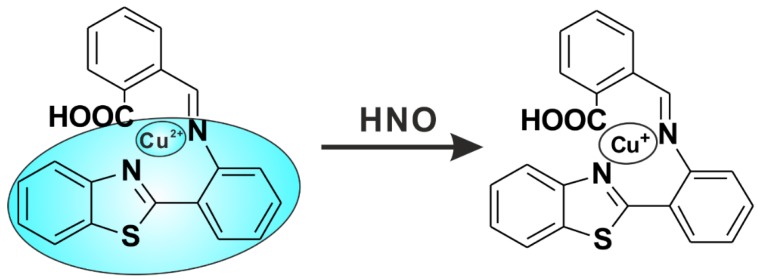
The proposed mechanism of fluorescence probe Cu(II)-AbTCA for HNO.

The photophysical properties of AbTCA and its copper complex Cu(II)-AbTCA as measured using PBS (10 mM, pH 7.4 containing 1% DMSO) are shown in Figures S8 and S9. The AbTCA absorption spectrum is broad, with two peaks at 284 and 358 nm (Figure S8). The absorption spectrum of Cu^2+^ added AbTCA is also broad with two peaks at 284 and 375 nm. In the fluorescence emission, AbTCA alone displayed negligible fluorescence at 450 nm (excited at 358 nm) whereas an evident increase in the fluorescence intensity was noticed at 450 nm after adding Cu^2+^ (Figure S9). In the design of AbTCA, it is believed that AbTCA has no individual binding sites and fluorophores and hence, the emission process was achieved by the whole molecule. Also, it is assumed that the obtained fluorescence increment was due to the avoidance of non-radiative relaxation process of nitrogen lone pair of electrons by Cu^2+^ binding and photoinduced electron transfer (PET) between the thiazole and 2-carboxybenzaldehyde moiety was blocked [41]. The formation of Cu(II)-AbTCA was utilized as a platform for selective HNO sensing. Furthermore, Angeli’s salt titration against Cu(II)-AbTCA reduced the transition metal ion from Cu(II) to Cu(I). The proposed mechanism for HNO sensing by fluorescence probe Cu(II)-AbTCA is shown in Scheme 1.

To further reveal the recognition pathway of Cu(II)-AbTCA for HNO, detailed investigations of Cu(II)-AbTCA with and without Angeli’s salt were conducted. From the fluorescence spectra results, it was found that Cu(II)-AbTCA reacts with Angeli’s salt with subsequent reduction in its fluorescence emission intensity. To confirm the Cu(I)-AbTCA formation is the only major product after the reaction of Cu(II)-AbTCA and HNO, the reaction mixture was further analyzed by ESI-MS, EPR, and cyclic voltammetry studies. The X-band EPR spectrum of Cu(II)-AbTCA complex measured at ambient temperature using THF, displayed an isotropic signal with a g value of 2.05 (Figure S10) which disappears upon Angeli’s salt treatment as expected for Cu(II) to Cu(I) reduction.

In addition, recorded ESI-MS spectra also confirmed the formation of Cu(I)-AbTCA. The spectra showed the disappearance of the original peak for Cu(II)-AbTCA and the formation of new peaks at *m*/*z* 445.7 and 478.1 corresponding to [(AbTCA)+Cu(I)+Na] and [(AbTCA)+Cu(I)+Cl+Na]^+^ (Figure S11). The electrochemical properties of Cu(II)-AbTCA and the ligand AbTCA were measured at a scan rate of 100 mVs^−1^. The results showed that the ligand AbTCA used in this study is neither reduced nor oxidized reversibly under the studied potential and this the redox processes can be exclusively assigned to copper ion. The electrochemical studies using cyclic voltammetry showed the quasi-reversible reduction at 230 mV for Cu(II)/Cu(I) (vs Fc/Fc^+^) (Figure S12). This evidence concluded that the Cu(I)-AbTCA was the only major product formed.

### 2.7. Detection of Nitroxyl Release from DNIC Complex, [PPN][Fe(NO)_4_] and Thiophenol

The red-brown solid, dinitrosyliron complex (DNIC), [PPN][Fe(NO)_4_] was synthesized and characterized as described in the reported literature by utilizing NO^+^ and [PPN][(S(CH_2_)_3_S)Fe(NO)_2_] complex in THF solution [42]. Of importance, the addition of two equivalents of thiophenol to [PPN][Fe(NO)_4_] generated the known compound [PPN][(PhS)_2_Fe(NO)_2_] along with HNO, as shown in Scheme S2. In order to ascertain whether Cu(II)-AbTCA could be used for detection of nitroxyl release from the DNIC complex, [PPN][Fe(NO)_4_] and thiophenol, we performed fluorescence measurements. Cu(II)-AbTCA (40 μM) originally showed high fluorescence intensity whereas upon treatment with thiophenol combined [PPN][Fe(NO)_4_] (200 μM), quenching of fluorescence (67%) was observed (Figure S13). These observations clearly revealed the turn-off behavior of Cu(II)-AbTCA thereby detection of nitroxyl release.

### 2.8. Cytotoxicity

Cytotoxicity studies were performed for the fluorescence probe Cu(II)-AbTCA to examine its potential toxicity. The toxicity of Cu(II)-AbTCA was investigated against EAHY-44926 and RAW 264.7 cells. To evaluate the cytotoxicity of Cu(II)-AbTCA, MTT assay was performed with various concentrations of Cu(II)-AbTCA against EAHY-44926 (0, 1.25, 2.5, 5, 10, and 20 µM) and RAW 264.7 (0, 1.25, 2.5, 5, 10 and 20 µM) cells for 24 h. The results obtained clearly showed that Cu(II)-AbTCA had low toxicity towards EAHY-44926 and RAW 264.7 cells under the experimental conditions (Figure S14).

### 2.9. Confocal Imaging

Compiled evidence from the previous studies prompted us to exploit the biological feasibility of Cu(II)-AbTCA as a fluorescence turn-off imaging probe. We intend to investigate the Cu(II)-AbTCA ability for HNO detection in EAHY-44926 cells and HNO detection in RAW 264.7 cells with the help of confocal fluorescence imaging.

#### 2.9.1. Detection of HNO Using EAHY-44926 Cells

**Figure 2:**
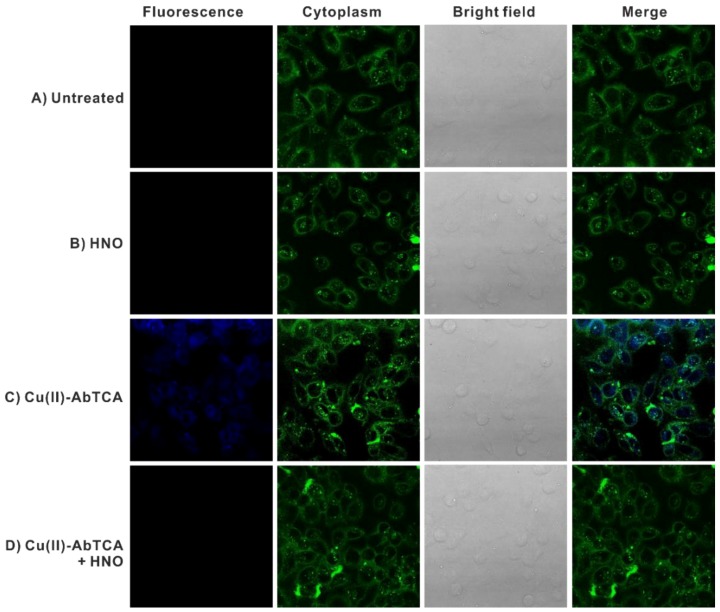
Confocal microscopy images of HNO in live EAHY-44926 cells as detected using Cu(II)-AbTCA (20 µM). Fluorescence images (**left**); Cytoplasm images (**middle**); Bright field images (**middle**); Merged images (**right**). (**A**) EAHY-44926 cells alone; (**B**) Cells treated with Angeli’s salt (200 µM); (**C**) Cells treated with Cu(II)-AbTCA; (**D**) Cells pretreated with Cu(II)-AbTCA followed by Angeli’s salt.

EAHY-44926 cells were incubated with Cu(II)-AbTCA (20 µM) for about 30 min and thoroughly washed to remove excess Cu(II)-AbTCA. Angeli’s salt (200 µM) was added and the cells were again kept for 30 min incubation. Control cells and cells with HNO did not show any fluorescence (Figure 2a,b). Treatment of EAHY-44926 cells with Cu(II)-AbTCA probe alone produced a strong fluorescence (Figure 2c). In contrast, Cu(II)-AbTCA treated EAHY-44926 cells incubated with HNO quenched the fluorescence indicating successful Cu(II) to Cu(I) reduction as well as the ON-OFF switching property of the probe.

#### 2.9.2. Detection of HNO Using RAW 264.7 Cells

Recently, it was found that the biological species such as ascorbate and tyrosine are capable of reducing NO to HNO. Imaging studies of HNO were also carried out by Lippard’s group. It was shown in their studies that the HNO was produced by pretreatment of cells with NO donor and then stimulating with sodium ascorbate (SA). Based on this, we further investigated whether Cu(II)-AbTCA can detect HNO formed via these mentioned biological process.

**Figure 3:**
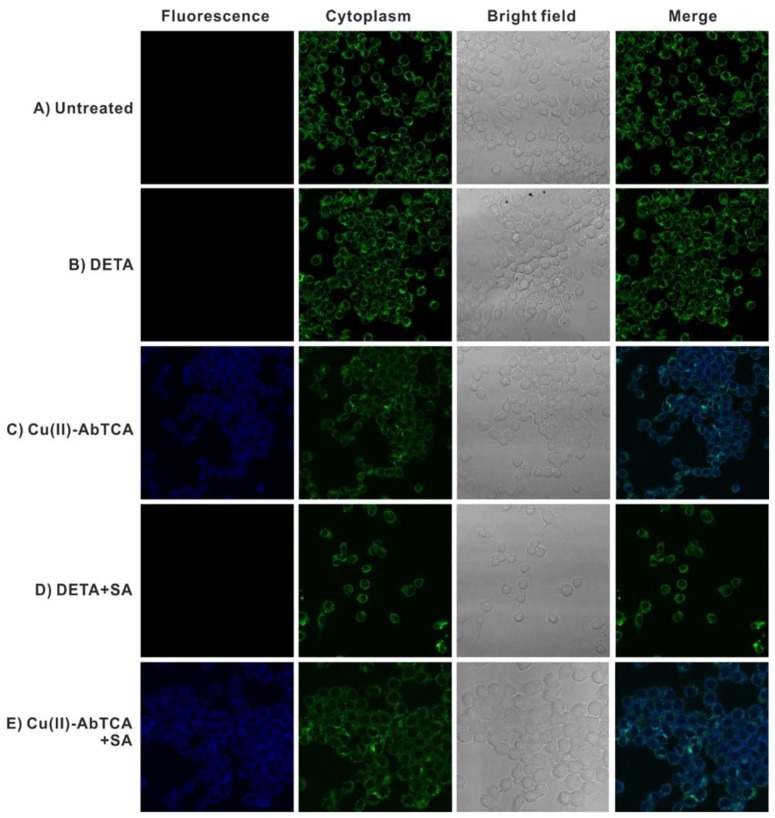
Confocal microscopy images of HNO in live RAW 264.7 cells as detected using Cu(II)-AbTCA (20 µM). Fluorescence images (**left**); Cytoplasm images (**middle**); Bright field images (**middle**); Merged images (**right**). (**A**) RAW 264.7 cells alone; (**B**) Cells treated with DETA NONOate; (**C**) Cells treated with Cu(II)-AbTCA; (**D**) Cells pretreated with Cu(II)-AbTCA followed by DETA NONOate and SA; (**E**) Cells pretreated with Cu(II)-AbTCA followed by SA.

In this study, imaging experiment was carried out with RAW 264.7 cells. Control cells and cells pretreated with DETA NONOate did not show any fluorescence (Figure 3a,b). Strong fluorescence was observed with Cu(II)-AbTCA treated RAW 264.7 cells whereas the reduction in the fluorescence was noticed for RAW 264.7 cells upon pre-treatment with DETA NONOate and sodium ascorbate (Figure 3c,d). In addition, co-localization studies were also performed to check the localization of the probe inside the cells. All the obtained experimental results clearly revealed that the majority of the Cu(II)-AbTCA is localized in the cell cytoplasm.

### 2.10. In Vivo Imaging of HNO in Zebrafish

**Figure 4:**
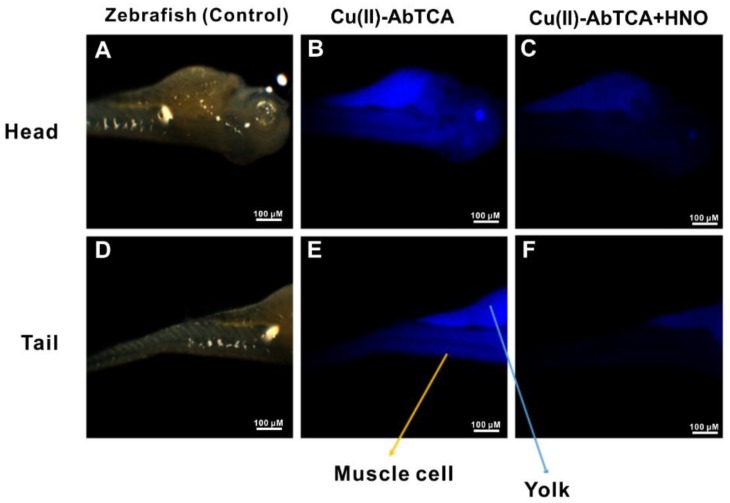
Fluorescence images of zebrafish. Head and tail portions of zebrafish alone (control) (**A**,**D**) zebrafish treated with Cu(II)-AbTCA (20 μM) for 40 min (**B**,**E**); zebrafish treated with Cu(II)-AbTCA (20 μM) for 40 min followed by addition of Angeli’s salt (200 μM) for 10 min (**C**,**F**).

The HNO production in both normal or failure canine heart is related with the increase of systolic force and hence we decided to investigate the Cu(II)-AbTCA ability to capture the image ingestion of HNO in zebrafish (*Danio rerio*), a vertebrate animal. Zebrafishes alone did not show any fluorescence whereas upon incubating these zebrafishes with Cu(II)-AbTCA (20 μM) for 10 min, intense fluorescence spots have appeared in the E3 embryo media (Figure 4). Further treatment of zebrafishes with Angeli’s salt concentration of about 200 µM for 10 min, an obvious reduction in the fluorescence intensity was observed in two areas around its head and tail portions. Muscle and yolk portions exhibited major effects whereas blood vessels and bone areas showed minor effects. Further, no deaths of zebrafishes were observed when exposed to Cu(II)-AbTCA (1 M) concentrations for 24 h, revealing its low toxic ability in the in vivo studies (data not shown). These results are fully consistent with the optical and imaging studies performed using the same probe. All these results substantiate that Cu(II)-AbTCA can behave as a highly sensitive turn-off fluorescence probe for HNO detection in vitro and in vivo.

## 3. Materials and Methods

### 3.1. Chemicals and Reagents

Cysteine (Cys), homocysteine (Hys), iron(III) chloride (FeCl3), hydrogen peroxide (H_2_O_2_), histidine (His), serine (Ser), arginine (Arg), isoleucine (Ile), alanine (Ala), lysine (Lys), asparagine (Asn), threonine (Thr), glutamine (Gln), leucine (Leu), proline (Pro), tryptophan (Trp), phenylalanine (Phe), S-nitrosoglutathione (RSNO), glutamic acid (Glu), peroxynitrite (ONOO^−^), 3-(4,5-dimethylthiazol-2-yl)-2,5-diphenyltetrazolium bromide reagent (MTT), dimethyl sulfoxide (DMSO), cetyltrimethyl ammonium bromide (CTAB), [N(PPh_3_)_2_] = PPN, 2-(2-aminophenyl)benzothiazole, and tetrabutylammonium hexafluorophosphate (n-Bu_4_NPF_6_) were purchased from Sigma Aldrich company (St. Louis, MO, USA). 2-Carboxybenzaldehyde was obtained from Alfa Aesar (Karlsruhe, Germany). Dulbecco’s minimal essential medium (DMEM), fetal bovine serum (FBS), 100× penicillin and streptomycin solution were ordered from Gibco (New York, NY, USA). Sodium hydrosulfide (NaSH) was purchased from Acros Organics (Morris, NJ, USA). Sodium nitrate (NaNO_3_)M and sodium nitrite (NaNO_2_) were acquired from Showa (Hirohito, Japan). DETA NONOate and sodium ascorbate were obtained from Caymann (Ann Arbor,, MI, USA). Copper(II) chloride dihydrate (CuCl_2_·2H_2_O) and the other salts used in this study were ordered from Riedel-de Haen (Hannover, Germany). All other chemicals and reagents used in this work were obtained from commercial suppliers.

### 3.2. Instrumentation

The ^1^H- and ^13^C-NMR spectra of the synthesized compounds were recorded in deuterated chloroform (CDCl_3_) using an INOVA 500 NMR spectrometer (Varian, Palo Alto, CA, USA). Proton and carbon chemical shifts were measured using tetramethylsilane (SiMe_4_ (δ = 0)) as an internal reference with respect to residual protons (δ = 2.5) and residual carbons (δ = 39.51) in the solvent, respectively. UV-visible and fluorescence spectra were recorded with a U-3010 UV-visible spectrophotometer (Hitachi, Tokyo, Japan) and F-9000 fluorescence spectrophotometer (Hitachi, Tokyo, Japan), respectively, at room temperature. Mass spectral data were acquired using an Autoflex II ESI mass spectrometer (Bruker, Billerica, MA, USA). A Lab 850 digital pH meter (SCHOTT, Mainz, Germany) was used to measure all the pH measurements. A CH Instruments (potentiostat, Austin, TX, USA) electrochemical analyzer was used to conduct cyclic voltammetric studies in acetonitrile using *n*-Bu_4_NPF_6_ as the supporting electrolyte. A three electrode set up comprising of glassy carbon, platinum, Ag/AgCl as working, auxiliary and reference electrodes, respectively was used. The nitrogen gas was purged in all the solutions before making measurements at room temperature. The HPLC separations were performed using an Waters 2695 HPLC instrument (Waters, Milford, MA, USA) with a multi-wavelength detector and automated fraction collector. A SUPELCO C18, 5 µm, 4.6 × 250 mm column (SUPELCO, Bellefonte, PA, USA) was used as a reverse stationary phase and a mixture of two solvents (A: 0.1% (*v*/*v*) trifluoroacetic acid (TFA) in H_2_O; B: 0.1% (*v*/*v*) TFA in MeOH) from 50% B to 100% B. The mobile phase used was acetonitrile-water (80:20) in a flow rate of 1 mL/min.

### 3.3. Chemistry

#### 3.3.1. Synthesis of 2-((2-(benzothiazole-2yl)benzylidene)amino)benzoic acid, AbTCA

To prepare AbTCA, 2-(2-aminophenyl)benzothiazole (500 mg, 2.21 mmol) and 2-carboxy-benzaldehyde (332 mg, 2.21 mmol) in ethanol were combined in a 100 mL round-bottomed flask (Scheme S1). The reaction mixture was refluxed at 80 °C for about 4 h. The precipitate was obtained and it was filtered, washed with water, and dried under vacuum. The spectral measurements were carried out without any purification. Yield: 563 mg (68%). ^1^H-NMR (CDCl_3_, 500 MHz): δ 10.12 (br d, 1H, OH), 6.9 (d, 1H, CH=N), 8.0 (d, 1H, Ar), 7.82–7.88 (m, 3H, Ar), 7.70–7.81 (m, 2H, Ar), 7.61–7.63 (m, 1H, Ar), 7.41–7.46 (m, 1H, Ar), 7.26–7.39 (m, 3H, Ar), 6.98–7.01 (m, 1H, Ar) ppm (Figure S1); ^13^C-NMR (CDCl_3_, 500 MHz): δ 169.37, 168.83, 153.00, 145.72, 144.12, 134.36, 133.13, 132.25, 130.73, 130.42, 128.07, 126.18, 125.79, 125.30, 122.96, 122.31, 121.26, 119.51, 116.97, 114.05, 86.72 ppm (Figure S2). ESI mass (positive mode, *m*/*z*): calculated: 358.08, found 359.2 for [M + 1] (Figure S3). HRESI-Mass (*m*/*z*) (C_21_H_15_N_2_O_2_S): Calculated: 358.0776, found 359.0849 for [M + 1] (Figure S4). The purity of AbTCA was determined to be >95% by analytical HPLC analysis (Figure S5).

#### 3.3.2. Synthesis of Cu(II)-AbTCA

Cu(II)-AbTCA was prepared by mixing AbTCA (500 mg, 0.97 mmol), and copper chloride (164 mg, 0.97 mmol) in a 50 mL round-bottomed flask using ethanol (50 mL) ass a solvent medium (Scheme S1). Then the reaction mixture was refluxed at 80 °C for about 4 h. The precipitate was obtained and it was filtered, washed with water, and dried under vacuum. The compound was utilized for further studies without subjecting to any purification. Yield: 153 mg (27%). HRESI-MS (positive mode, *m*/*z*): calculated: 492.43, found 514.98 for [M + Na]^+^ (Figure S6).

### 3.4. Fluorimetry Analysis

Parent stock solutions of AbTCA (1 mM) and Cu(II)-AbTCA (1mM) were prepared by dissolving appropriate quantity of these substances in dimethyl sulfoxide. The stock solution of Angeli’s salt was prepared based on the reported literature [1]. Stock solutions of all the test analytes were prepared using distilled water. For typical optical measurements, Cu(II)-AbTCA was diluted to 40 μM by adding appropriate aliquots from stock solution, and then diluted for 2 mL using PBS (10 mM, pH 7.4 containing 1% DMSO and 0.01 M CTAB). The UV-visible and fluorescence spectra were then recorded upon addition of HNO and each spectral data were read after 5 min of HNO addition. All the spectra were obtained in a quartz cuvette of 2 cm path length and all the measurements were recorded at 25 °C.

The fluorescence quantum yield (ϕF) was determined by using quinine sulfate (ϕF = 0.58 in 0.1 M H_2_SO_4_ aqueous solution as the fluorescence standard [43,44]. The quantum was calculated using the following equation:

(1) ΦF(X) = ΦF(S) (ASFX/AXFS) (nX/nS)2

where *ΦF* is the fluorescence quantum yield, *A* is the absorbance at the excitation wavelength, *F* is the area under the corrected emission curve, and *N* is the refractive index of the solvent used. The subscripts *S* and *X* refers to the standard and to the unknown, respectively.

### 3.5. Determination of the Detection Limit

The linear relationship between the fluorescence intensity and the concentration of HNO was fitted based on the fluorescence titration. The detection limit was calculated using the following equation based on the fluorescence titration [4]:

(2) Detection limit = 3σ/S

where *σ* is the standard deviation of the blank sample and *S* is the slope of the linear regression equation.

### 3.6. Cell Culture and Cytotoxicity

EAHY-44926 and RAW 264.7 cells were purchased from the Food Industry Research and Development Institute (FIRDI, Hsinchu City, Taiwan) and American Type Culture Collection (Manassas, VA, USA), respectively. The cells were cultured in DMEM supplemented with 10% FBS and appropriate amounts of penicillin and streptomycin in a humidified incubator at 37 °C and 5% CO_2_. For cytotoxicity experiments, cells were seeded in 24-well plate at 1 × 104 cells/well density for 24 h. The test compound Cu(II)-AbTCA was added at desired strengths (0, 1.25, 2.5, 5, 10 and 20 µM). The culture supernatants were discarded and then cells were washed thrice using PBS and finally estimated the cell viability using MTT assay. For this, MTT reagent (100 µL) solution was added to each well of microplate, incubated for 2 h, and then treated with DMSO (50 µL) for 3–5 min. The absorption at 570 nm was measured using plate reader and the data were represented with mean ± SD for three separate experiments.

#### 3.6.1. Confocal Microscopy

The EAHY-44926 and RAW 264.7 cells used in confocal studies were cultured using DMEM medium supplemented with 10% FBS and an appropriate quantity of penicillin and streptomycin. The cells were seeded at a density of 1 × 10^4^ cells in confocal dishes containing 2 mL of medium at 37 °C and allowed to adhere to the dish for next 24 h. After 24 h, the medium was removed and the cells were again washed twice using DMEM. The cells were then incubated with Cu(II)-AbTCA (20 µM) for 30 min and further washed twice using PBS to remove excess Cu(II)-AbTCA in the medium. Subsequently, Angeli’s salt (0 and 200 µM) was added to the dish and further incubated for 1 h. The cells were washed using DMEM after incubation and before imaging. A TCS-SP5-X AOBS confocal scanning microscope (Leica, Wetzlar, Germany) was used to capture the fluorescence images at λ_ex_ = 375 nm and λ_em_ = 450 nm. For the co-localization experiments, Hylite Fluo 488 was used for the staining of the cytoplasm. Hylite Fluo 488 was imaged at λ_ex_ = 502 nm and λ_em_ = 517–537 nm.

#### 3.6.2. HNO imaging Studies

A study reported by Lippard’s group showed that HNO is endogenously produced upon pretreating cells with NO donor and then further stimulated with sodium ascorbate [31]. Diethylenetriamine NONOate (DETA NONOate) is a NO donor. In this experiment, NO was generated from DETA NONOate (1 mM stock solution in 10 mM NaOH). Approximately, 1 × 10^4^ cells were seeded in 8-well ibidi plates, allowed to adhere for 4 h and pre-incubated with DETA NONOate (final concentration 200 µM) for 20 h in DMEM and washed using PBS. The cells were subjected to imaging in PBS before being treated with Cu(II)-AbTCA (final concentration 20 µM) for about 45 min. The cells were washed with PBS once for 45 min. The cells were once again washed with PBS and set to take another set of images. Followed by this, sodium L-ascorbate (final concentration of 1.5 mM) was added and incubated for 30 min. The Leica TCS-SP5-X AOBS confocal scanning microscope was used to capture the fluorescence images at λ_ex_ = 375 nm and λ_em_ = 450 nm.

#### 3.6.3. Zebrafish Imaging Studies

Zebrafishes used in this study were maintained in E3 embryo media (15 mM NaCl, 0.5 mM KCl, 1 mM MgSO_4_, 1 mM CaCl_2_, 0.15 mM KH_2_PO_4_, 0.05 mM Na_2_HPO_4_, 0.7 mM NaHCO_3_, 5–10% methylene blue, pH 7.5) at 28 °C. To record fluorescence imaging in this study, 5–8 days old zebrafishes were incubated with Cu(II)-AbTCA (20 μM) in E3 embryo media for 20 min and washed with PBS to remove excess Cu(II)-AbTCA. After washing, Angeli’s salt (200 µM) solution was added to the zebrafishes and kept for 10 min incubation. The experiment was approved by institutional review board of National Chiao Tung University. A Leica TCS-SP5-X AOBS confocal scanning microscope was used to capture the fluorescence images at an excitation wavelength of 375 nm through 10 × 0.4 NA objective for zebrafishes. The fluorescence images were recorded in the range of 500–540 nm.

## 4. Conclusions

In this study, a new turn-off fluorescence probe Cu(II)-AbTCA for selective detection of HNO using different experimental methods was reported. The probe was fluorescent in its free form but the fluorescence emission was reduced upon specifically reacting with Angeli’s salt. The Cu(II)-AbTCA was highly selective for HNO over other tested biological competitors including biomolecules, anions and particularly the HNO analog NO. The calculated detection limit was 9.05 µM. The Cu(II)-AbTCA exhibited a fast response time of 10 s, and it is relatively easy to synthesize. To date, this is the first report mentioning the detection of nitroxyl release from DNIC complex, [PPN][Fe(NO)_4_] and thiophenol using Cu(II)-AbTCA. In addition, Cu(II)-AbTCA showed low cytotoxicity and could be successfully used for HNO detection using living EAHY-44926 and RAW 264.7 cells, respectively as well as in zebrafish. In particular, Cu(II)-AbTCA was noticed to be mostly localized in the cell cytoplasm indicating that Cu(II)-AbTCA might be very useful for HNO imaging in the cytoplasm. We also expect that this Cu(II)-AbTCA turn-off fluorescence probe will provide great benefits to biomedical applications using HNO.

## Supplementary Materials

The following are available online. Figure S1: ^1^H-NMR spectrum of AbTCA in CDCl_3_ at 400 MHz, Figure S2: ^13^C-NMR spectrum of AbTCA in CDCl_3_ at 100 MHz, Figure S3: ESI-Mass spectrum of AbTCA, Figure S4: HRESI-Mass spectrum of AbTCA, Figure S5: Analytical HPLC traces of AbTCA, Figure S6: HRESI-Mass spectrum of Cu(II)-AbTCA, Figure S7: Interference study of Cu(II)-AbTCA with other metal ions, Figure S8: Absorption spectra of AbTCA (red line) and Cu^2+^ added AbTCA(black line) in PBS (10 mM, pH 7.4 containing 1% DMSO), Figure S9: Fluorescence spectra of AbTCA and Cu^2+^ added AbTCA in PBS (10 mM, pH 7.4 containing 1% DMSO), Figure S10: EPR spectra recorded at 298 K for 40 μM Cu(II)-AbTCA (black line) and with excess Angeli’s salt (red line), Figure S11: ESI-Mass spectrum of HNO treated Cu(II)-AbTCA, Figure S12: Cyclic voltammograms of AbTCA and Cu(II)-AbTCA, Figure S13: Detection of nitroxyl release from DNIC complex, [PPN][Fe(NO)_4_] and thiophenol using Cu(II)-AbTCA probe, Figure S14: Evaluation of the potential cytotoxicity of Cu(II)-AbTCA to (A) EAHY-44926 cells; (B) RAW 264.7 cells, Scheme S1: Synthetic route of AbTCA and Cu(II)-AbTCA, Scheme S2: Generation of nitroxyl from dinitrosyliron complex (DNIC), [PPN][Fe(NO)_4_] and thiophenol.
