# Supplementary material for: In Vitro and in Vivo Imaging of Nitroxyl with Copper Fluorescent Probe in Living Cells and Zebrafish
Source: Molecules. 2018 Oct 6;23(10):2551. doi: 10.3390/molecules23102551 (PMC6222915; doi:10.3390/molecules23102551)
Supplement: Supplementary file 1 [file molecules-23-02551-s001.pdf]

In vitro and *in vivo* imaging of nitroxyl with Copper fluorescent probe in living cells and zebrafish

Sathyadevi Palanisamy [1,†], Yu-Liang Wang [1,†], Yu-Jen Chen [1], Chiao-Yun Chen [2,3], Fu-Te Tsai [4], Wen-Feng Liaw [4,] and Yun-Ming Wang [1,5,*]

[1] Department of Biological Science and Technology, Institute of Molecular Medicine and Bioengineering, Center For Intelligent Drug Systems and Smart Bio-devices (IDS2B), National Chiao Tung University, 75 Bo-Ai Street, Hsinchu 300, Taiwan; sathyadevi85@gmail.com (S.P.); littlestars2@livemail.tw (Y.-L.W); c2h5oh.eric@gmail.com (Y.-J.C)
[2] Department of Radiology, Faculty of Medicine, College of Medicine, Kaohsiung Medical University, Kaohsiung 807, Taiwan; ccy0103@hotmail.com
[3] Department of Medical Imaging, Kaohsiung Medical University Hospital, Kaohsiung 807, Taiwan
[4] Department of Chemistry, National Tsing Hua University, Hsinchu 30043, Taiwan; fttsai@mx.nthu.edu.tw (F.-T.T.); wfliaw@mx.nthu.edu.tw (W.-F.L.)
[5] Department of Biomedical Science and Environmental Biology, Kaohsiung Medical University, Kaohsiung 807, Taiwan

*Correspondence: ymwang@mail.nctu.edu.tw; Tel.: +886-3-5712121 ext. 56972, Fax: +886-3-5729288

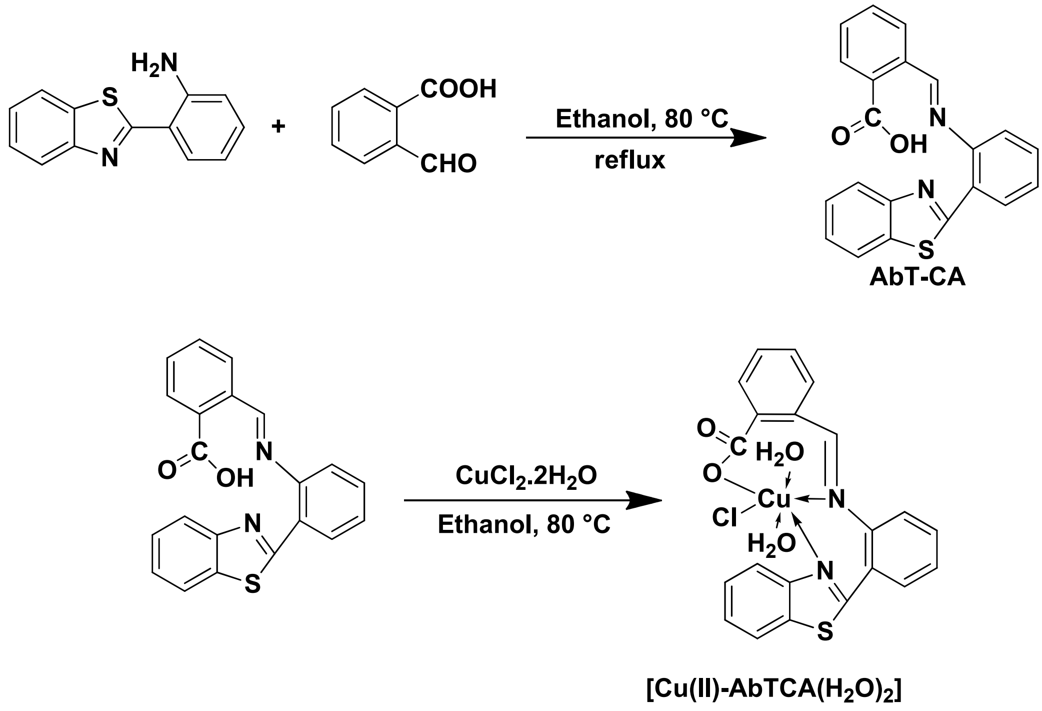

Scheme S1. Synthetic route of AbTCA and Cu(II)-AbTCA.

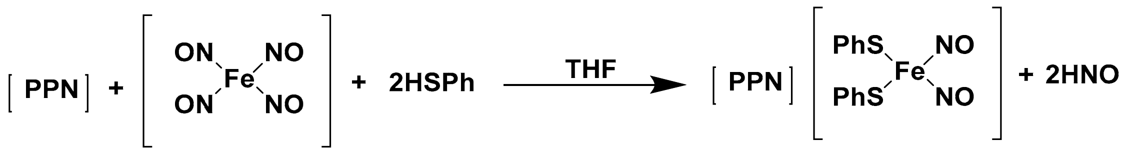

Scheme S2. Generation of nitroxyl from dinitrosyliron complex (DNIC), [PPN][Fe(NO)$_4$] and thiophenol.

(a)

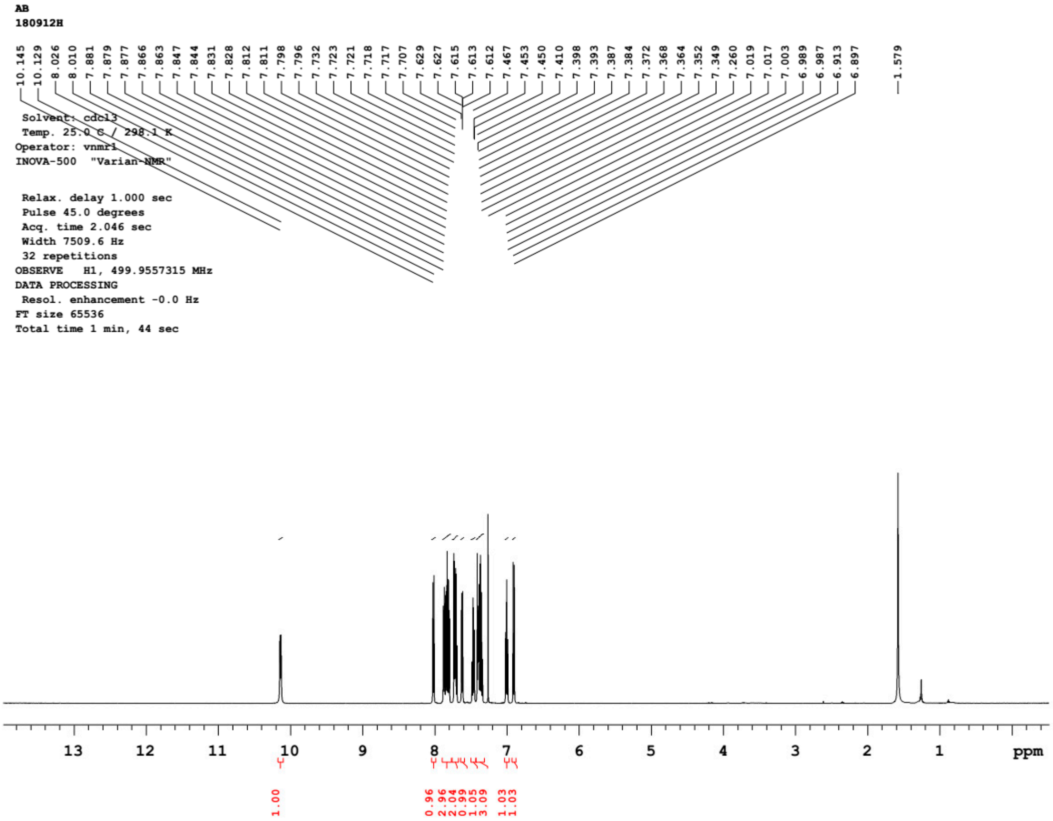
AB
180912H
10.145 10.129 8.026 8.010 7.881 7.879 7.877 7.866 7.863 7.847 7.844 7.831 7.828 7.812 7.811 7.798 7.796 7.732 7.723 7.721 7.718 7.717 7.707 7.629 7.627 7.615 7.613 7.612 7.467 7.453 7.450 7.410 7.398 7.393 7.387 7.384 7.372 7.368 7.364 7.352 7.349 7.260 7.019 7.017 7.003 6.989 6.987 6.913 6.897 1.579
Solvent: cdcl3
Temp. 25.0 C / 298.1 K
Operator: vnmr1
INOVA-500 "Varian-NMR"
Relax. delay 1.000 sec
Pulse 45.0 degrees
Acq. time 2.046 sec
Width 7509.6 Hz
32 repetitions
OBSERVE H1, 499.9557315 MHz
DATA PROCESSING
Resol. enhancement -0.0 Hz
FT size 65536
Total time 1 min, 44 sec
13 12 11 10 9 8 7 6 5 4 3 2 1 ppm
1.00 0.96 2.96 2.04 0.99 1.05 3.09 1.03 1.03

(b)

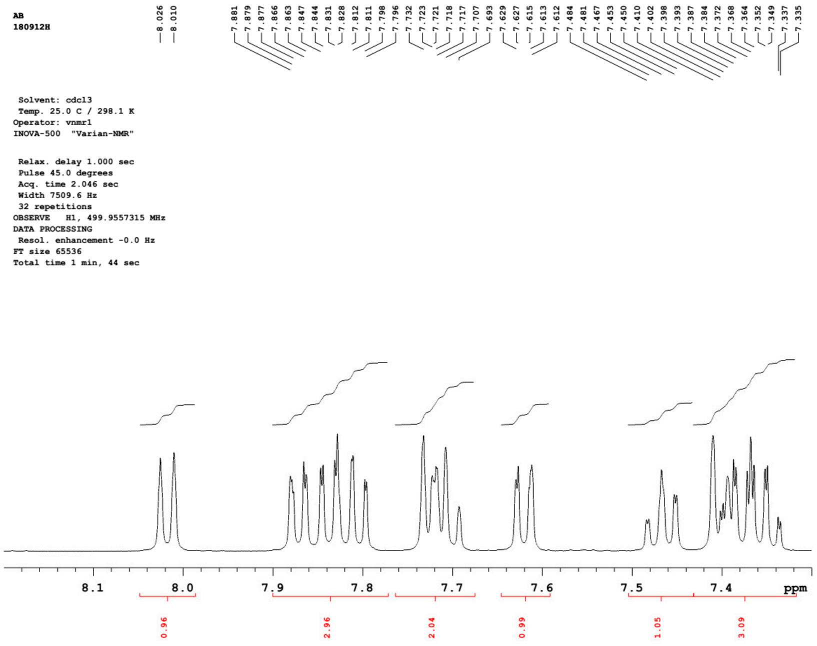
AB
180912H
8.026 8.010 7.881 7.879 7.877 7.866 7.863 7.847 7.844 7.831 7.828 7.812 7.811 7.798 7.796 7.732 7.723 7.721 7.718 7.717 7.707 7.693 7.629 7.627 7.615 7.613 7.612 7.484 7.481 7.467 7.453 7.450 7.410 7.402 7.398 7.393 7.387 7.384 7.372 7.368 7.364 7.352 7.349 7.337 7.335
Solvent: cdcl3
Temp. 25.0 C / 298.1 K
Operator: vnmr1
INOVA-500 "Varian-NMR"
Relax. delay 1.000 sec
Pulse 45.0 degrees
Acq. time 2.046 sec
Width 7509.6 Hz
32 repetitions
OBSERVE H1, 499.9557315 MHz
DATA PROCESSING
Resol. enhancement -0.0 Hz
FT size 65536
Total time 1 min, 44 sec
8.1 8.0 7.9 7.8 7.7 7.6 7.5 7.4 ppm
0.96 2.96 2.04 0.99 1.05 3.09

(c)

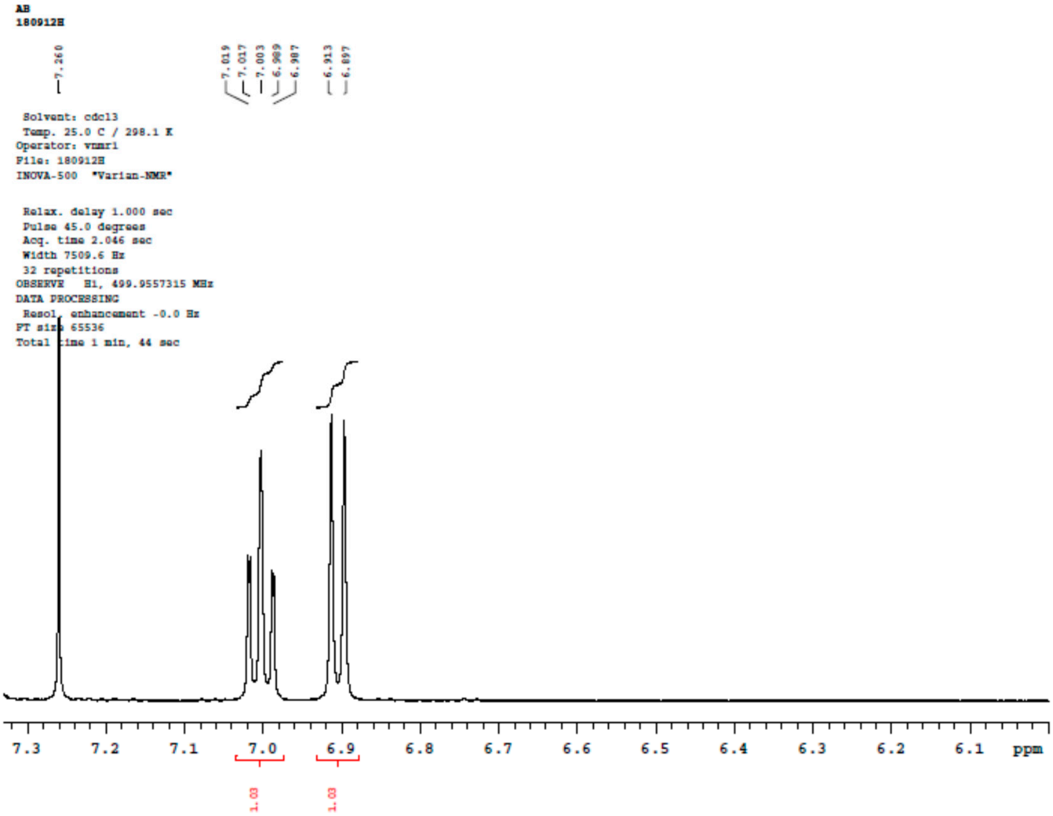
AB
180912H
7.260
7.019
7.017
7.003
6.989
6.987
6.913
6.897
Solvent: cdcl3
Temp. 25.0 C / 298.1 K
Operator: vnmr1
File: 180912H
INOVA-500 "Varian-NMR"
Relax. delay 1.000 sec
Pulse 45.0 degrees
Acq. time 2.046 sec
Width 7509.6 Hz
32 repetitions
OBSERVE H1, 499.9557315 MHz
DATA PROCESSING
Resol. enhancement -0.0 Hz
FT si[illegible] 65536
Total time 1 min, 44 sec
ppm
1.03
1.03

(d)

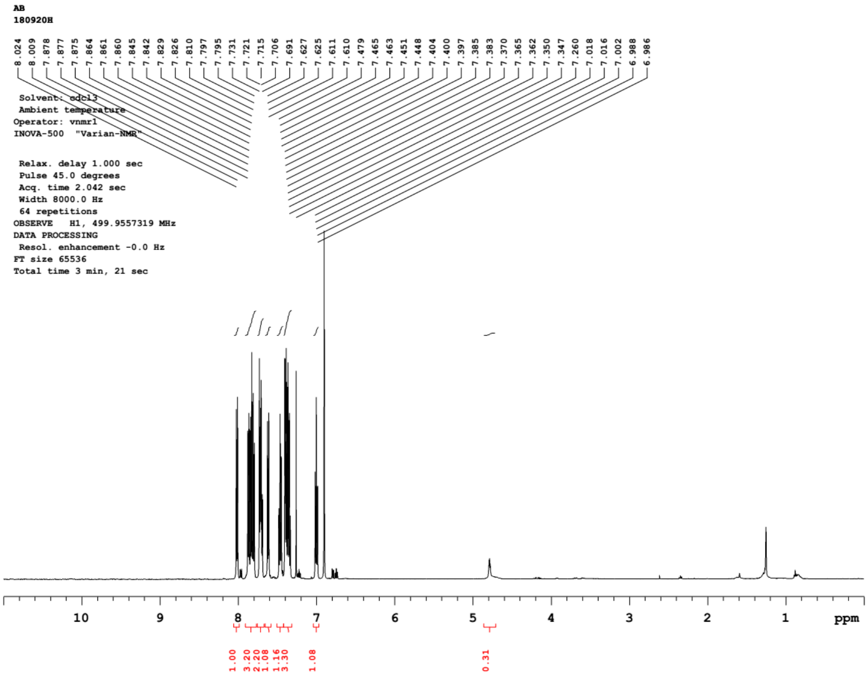
AB
180920H
8.024
8.009
7.878
7.877
7.875
7.864
7.861
7.860
7.845
7.842
7.829
7.826
7.810
7.797
7.795
7.731
7.721
7.715
7.706
7.691
7.627
7.625
7.611
7.610
7.479
7.465
7.463
7.451
7.448
7.404
7.400
7.397
7.385
7.383
7.370
7.365
7.362
7.350
7.347
7.260
7.018
7.016
7.002
6.988
6.986
Solvent: cdcl3
Ambient temperature
Operator: vnmr1
INOVA-500 "Varian-NMR"
Relax. delay 1.000 sec
Pulse 45.0 degrees
Acq. time 2.042 sec
Width 8000.0 Hz
64 repetitions
OBSERVE H1, 499.9557319 MHz
DATA PROCESSING
Resol. enhancement -0.0 Hz
FT size 65536
Total time 3 min, 21 sec
ppm
1.00
3.20
2.20
1.08
1.16
3.30
1.08
0.31

(e)

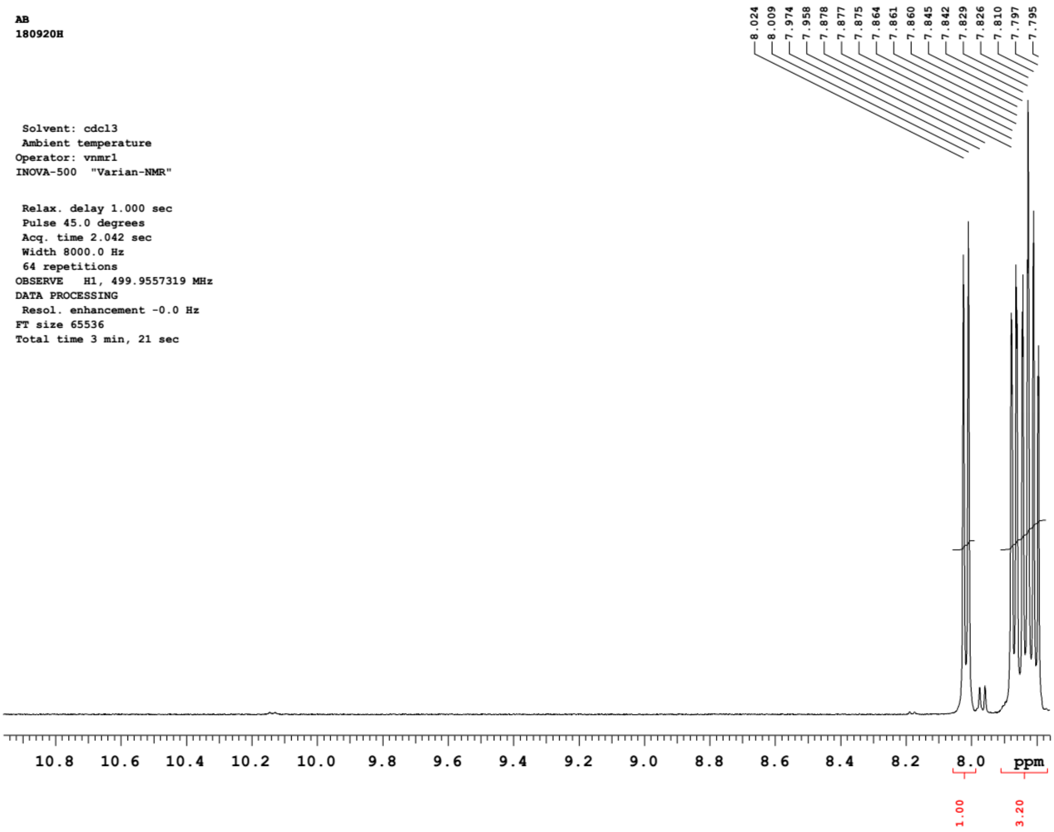
AB
180920H
Solvent: cdcl3
Ambient temperature
Operator: vnmr1
INOVA-500 "Varian-NMR"
Relax. delay 1.000 sec
Pulse 45.0 degrees
Acq. time 2.042 sec
Width 8000.0 Hz
64 repetitions
OBSERVE H1, 499.9557319 MHz
DATA PROCESSING
Resol. enhancement -0.0 Hz
FT size 65536
Total time 3 min, 21 sec
8.024 8.009 7.974 7.958 7.878 7.877 7.875 7.864 7.861 7.860 7.845 7.842 7.829 7.826 7.810 7.797 7.795
10.8 10.6 10.4 10.2 10.0 9.8 9.6 9.4 9.2 9.0 8.8 8.6 8.4 8.2 8.0 ppm
1.00 3.20

(f)

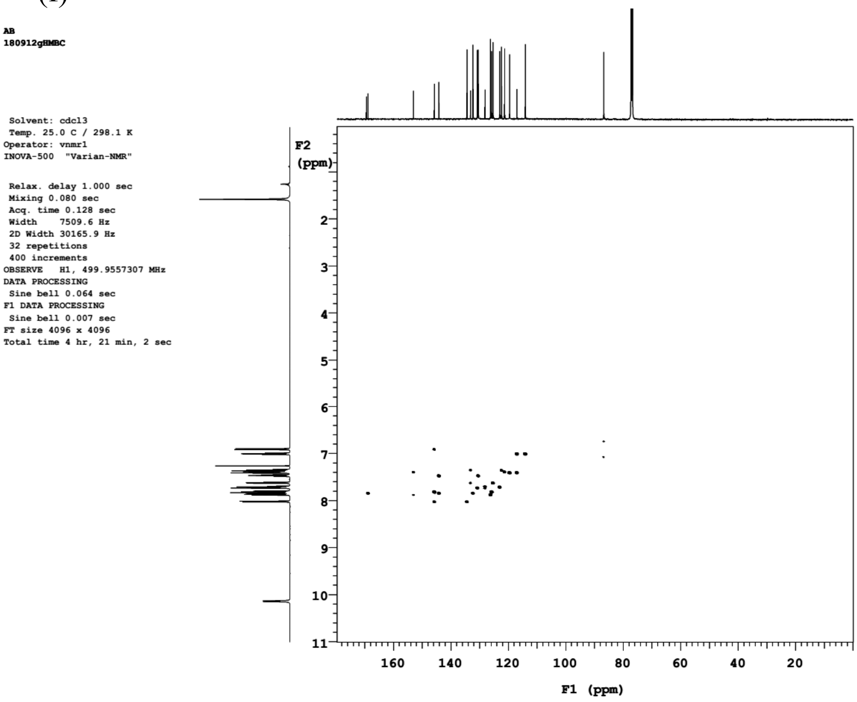
AB
180912gHMBC
Solvent: cdcl3
Temp. 25.0 C / 298.1 K
Operator: vnmr1
INOVA-500 "Varian-NMR"
Relax. delay 1.000 sec
Mixing 0.080 sec
Acq. time 0.128 sec
Width 7509.6 Hz
2D Width 30165.9 Hz
32 repetitions
400 increments
OBSERVE H1, 499.9557307 MHz
DATA PROCESSING
Sine bell 0.064 sec
F1 DATA PROCESSING
Sine bell 0.007 sec
FT size 4096 x 4096
Total time 4 hr, 21 min, 2 sec
F2 (ppm)
2 3 4 5 6 7 8 9 10 11
160 140 120 100 80 60 40 20
F1 (ppm)

(g)

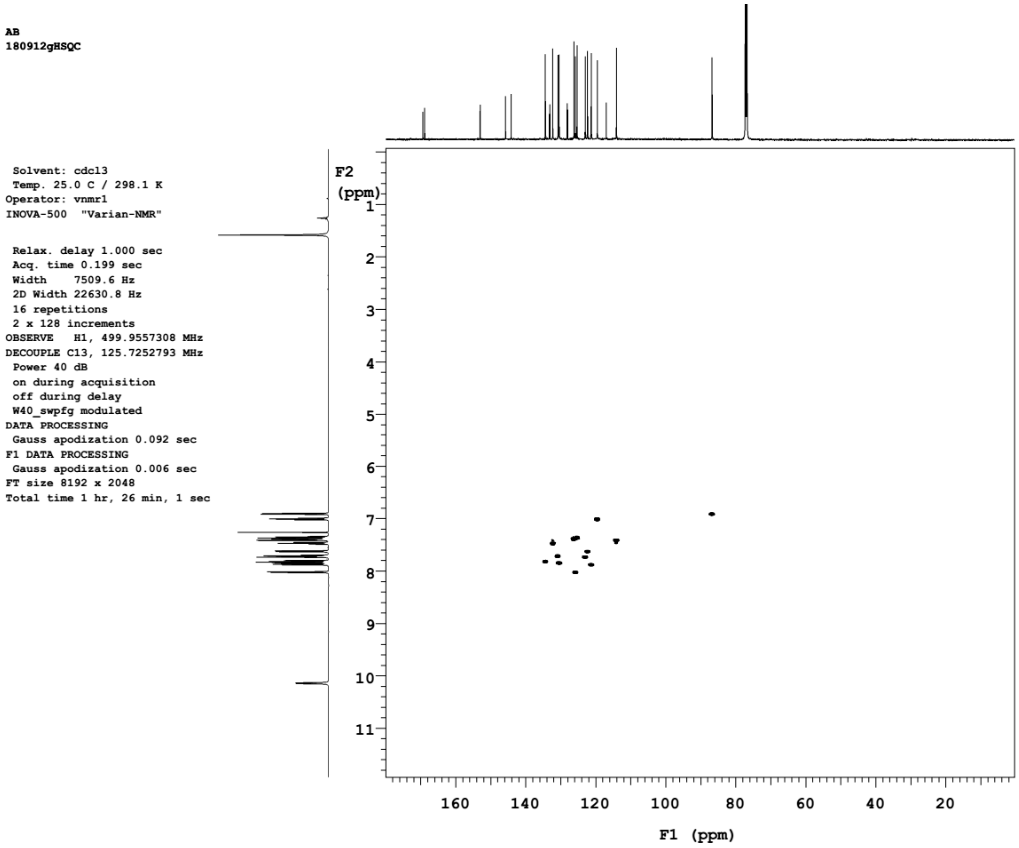
AB
180912gHSQC
Solvent: cdcl3
Temp. 25.0 C / 298.1 K
Operator: vnmr1
INOVA-500 "Varian-NMR"
Relax. delay 1.000 sec
Acq. time 0.199 sec
Width 7509.6 Hz
2D Width 22630.8 Hz
16 repetitions
2 x 128 increments
OBSERVE H1, 499.9557308 MHz
DECOUPLE C13, 125.7252793 MHz
Power 40 dB
on during acquisition
off during delay
W40_swpfg modulated
DATA PROCESSING
Gauss apodization 0.092 sec
F1 DATA PROCESSING
Gauss apodization 0.006 sec
FT size 8192 x 2048
Total time 1 hr, 26 min, 1 sec
F2 (ppm)
F1 (ppm)

(h)

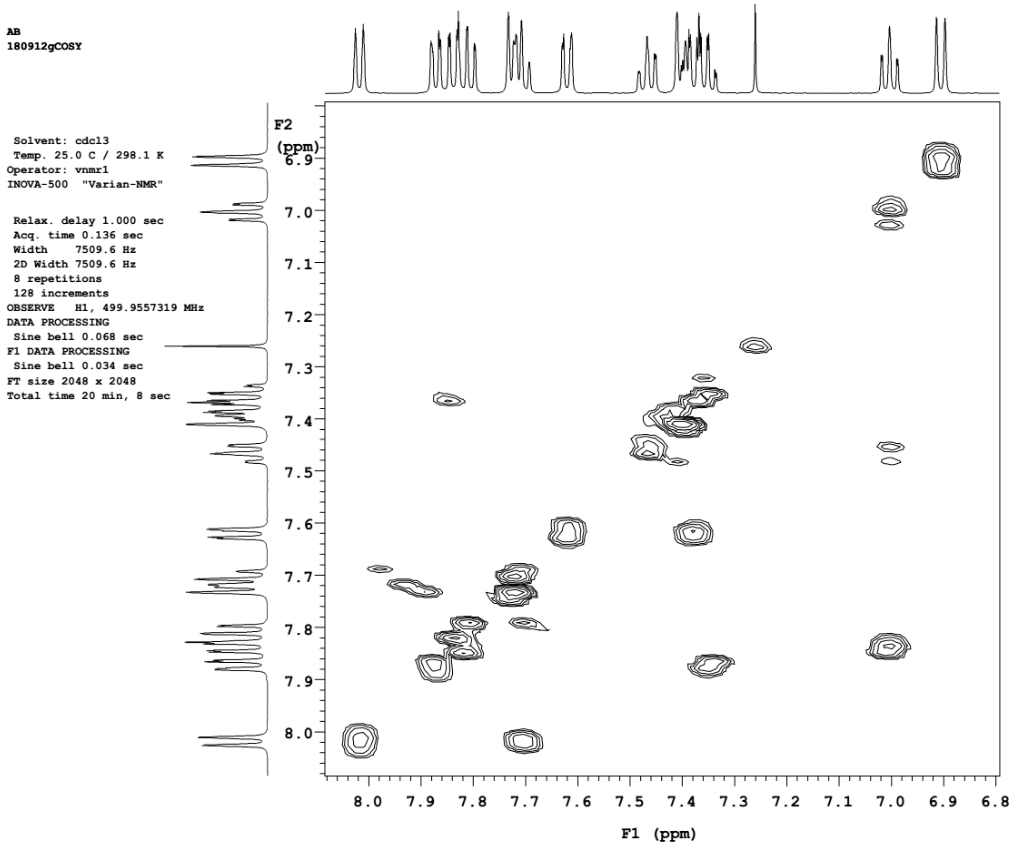

Figure S1. (a) ^{1}H NMR spectrum of AbTCA ($CDCl_3$, 500 MHz); (b,c) Expansion of ^{1}H NMR spectrum; (d,e) D_2O exchange of ^{1}H NMR spectrum and expansion; (f) gHMBC 2D NMR spectrum of AbTCA; (g) gHSQC 2D NMR spectrum of AbTCA; (h) gCOSY 2D NMR spectrum of AbTCA.

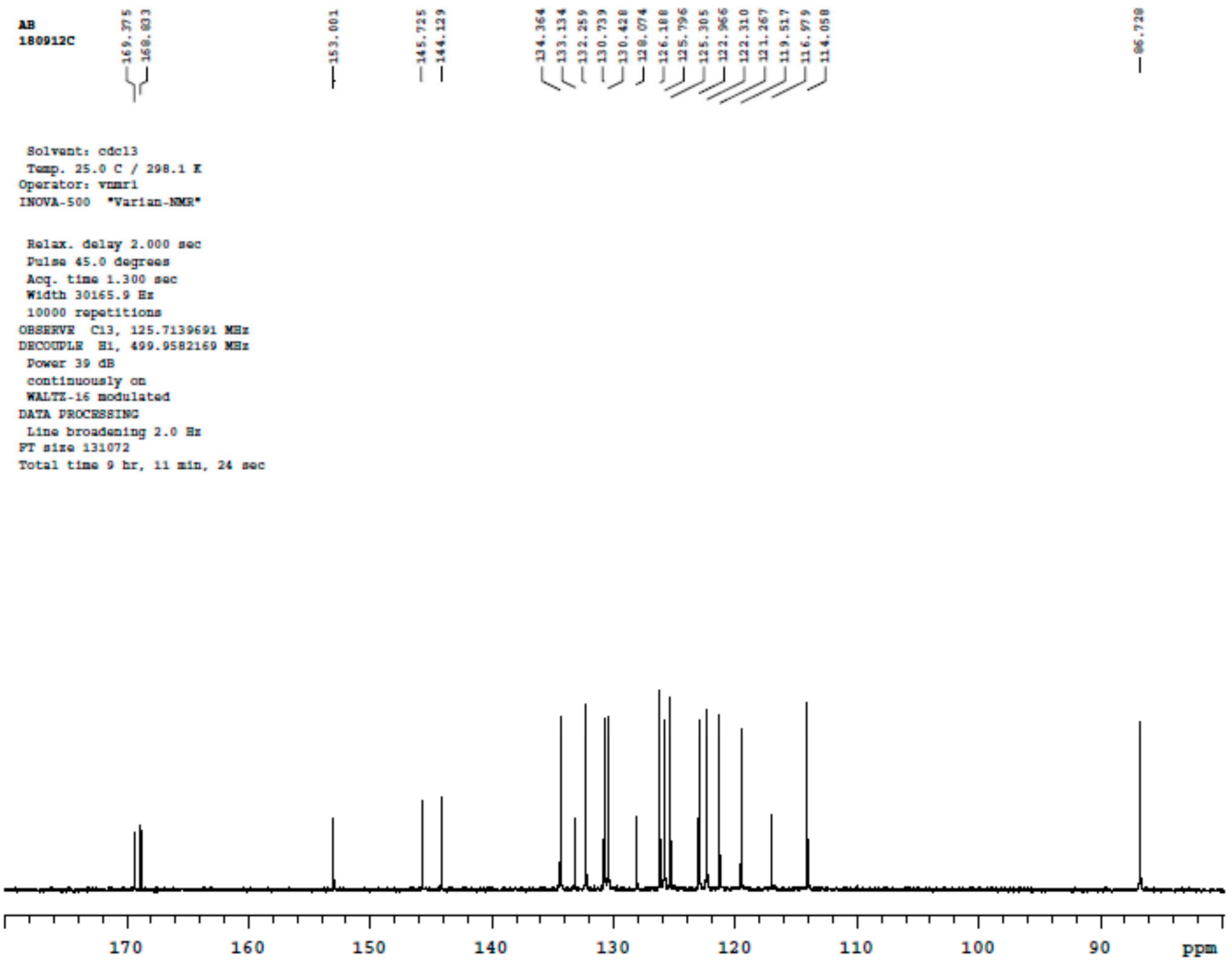

Figure S2. ^{13}C NMR spectrum of AbTCA in $CDCl_3$.

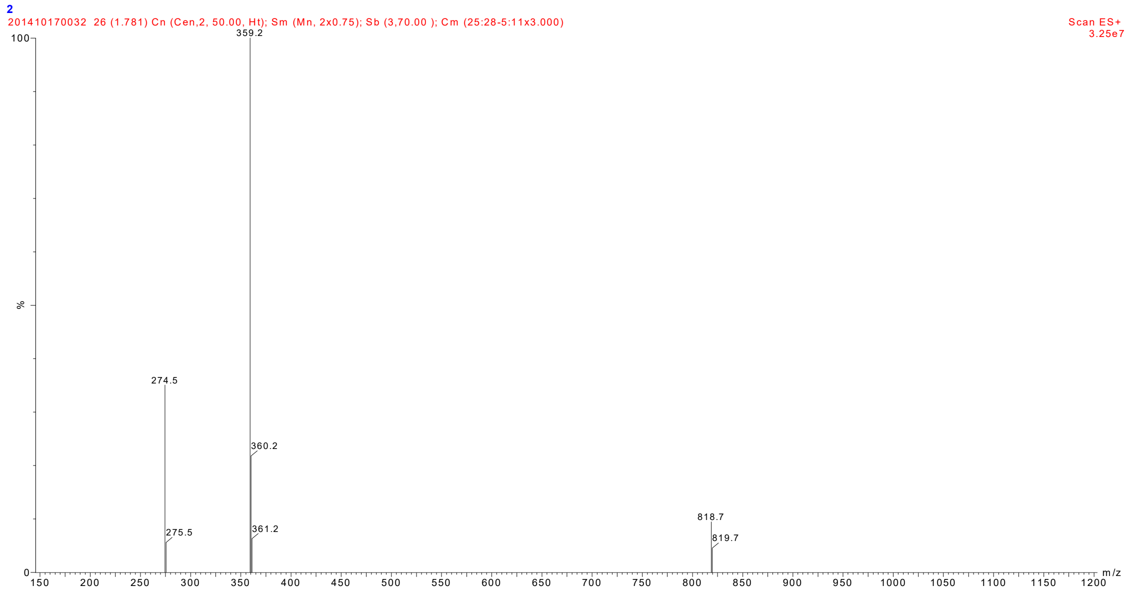

Figure S3. ESI-Mass spectrum of AbTCA.

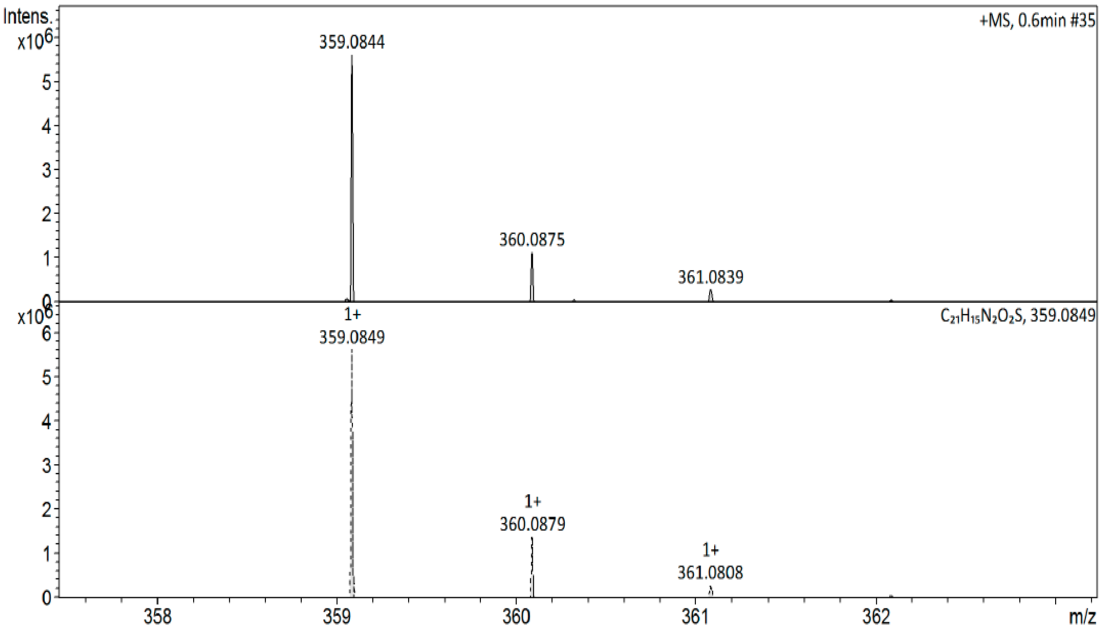

Figure S4. HRESI-Mass spectrum of AbTCA.

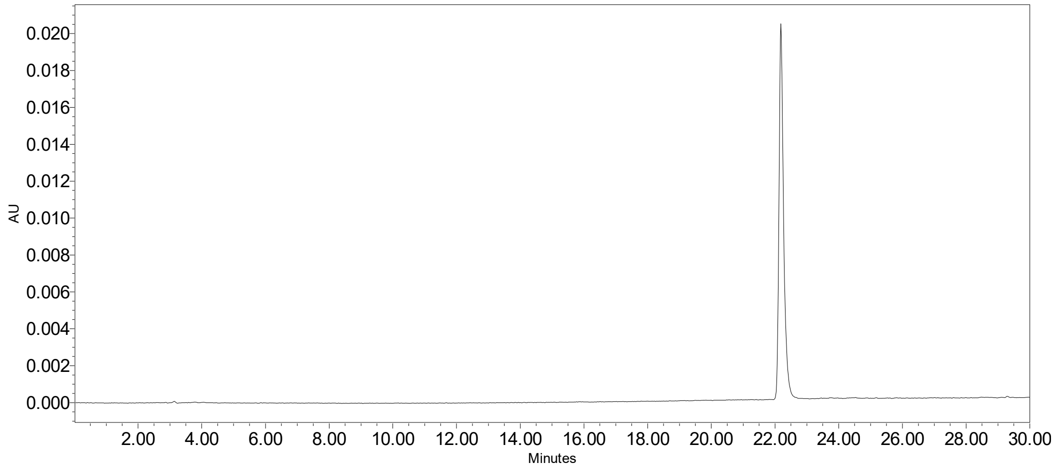

Figure S5. Analytical HPLC traces of AbTCA.

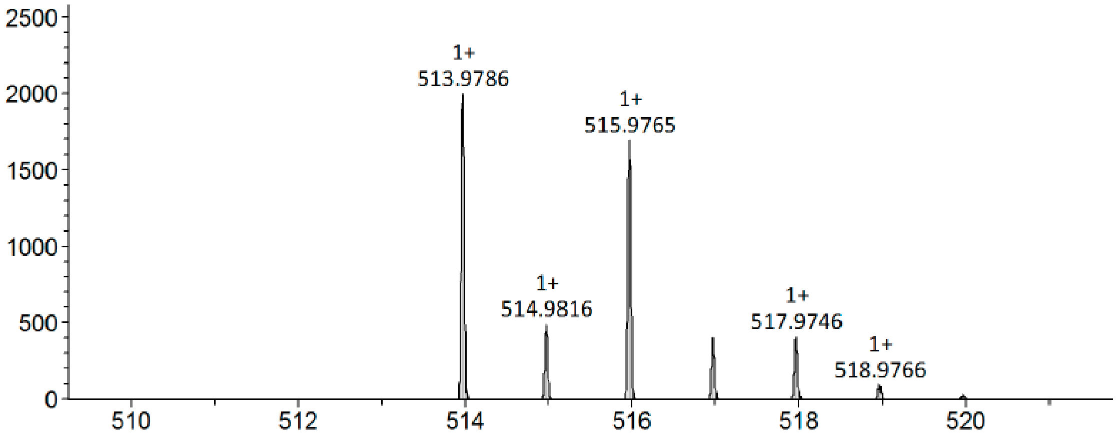

Figure S6. HRESI-Mass spectrum of Cu(II)-AbTCA.

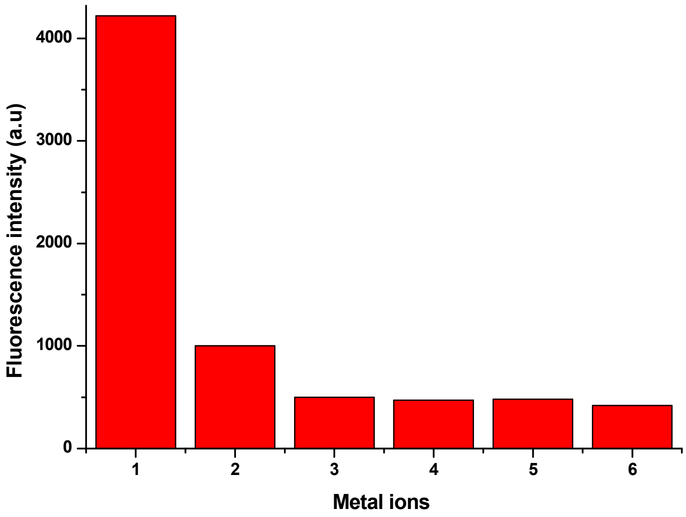

Figure S7. Fluorescence response of AbTCA added (1) Cu^{2+} (40 µM); (2) Zn^{2+} (100 µM); (3) Mg^{2+} (100 µM); (4) Co^{2+} (100 µM); (5) Cu^{+} (100 µM); and (6) Ni^{2+} (100 µM) in PBS (10 mM, pH 7.4 containing 1% DMSO).

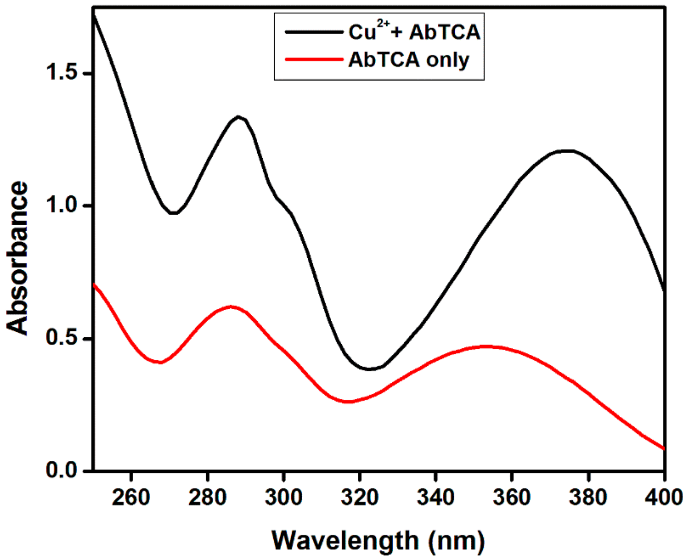

Figure S8. Absorption spectra of AbTCA (red line) and Cu^{2+} added AbTCA(black line) in PBS (10 mM, pH 7.4 containing 1% DMSO).

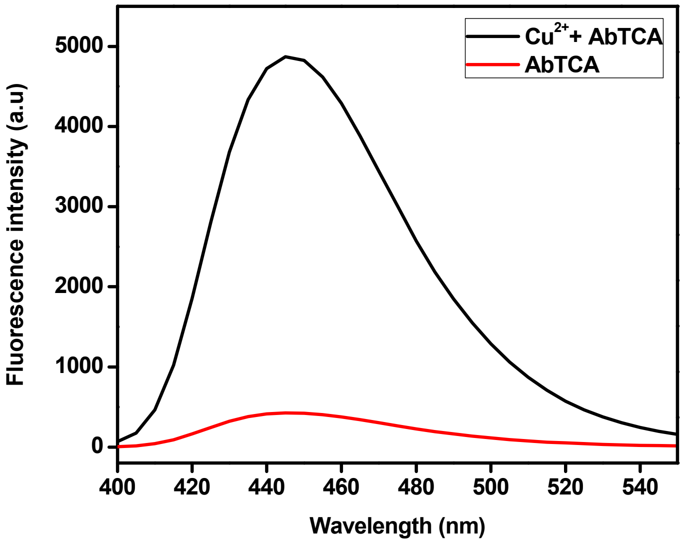

Figure S9. Fluorescence spectra of AbTCA and Cu^{2+} added AbTCA in PBS (10 mM, pH 7.4 containing 1% DMSO).

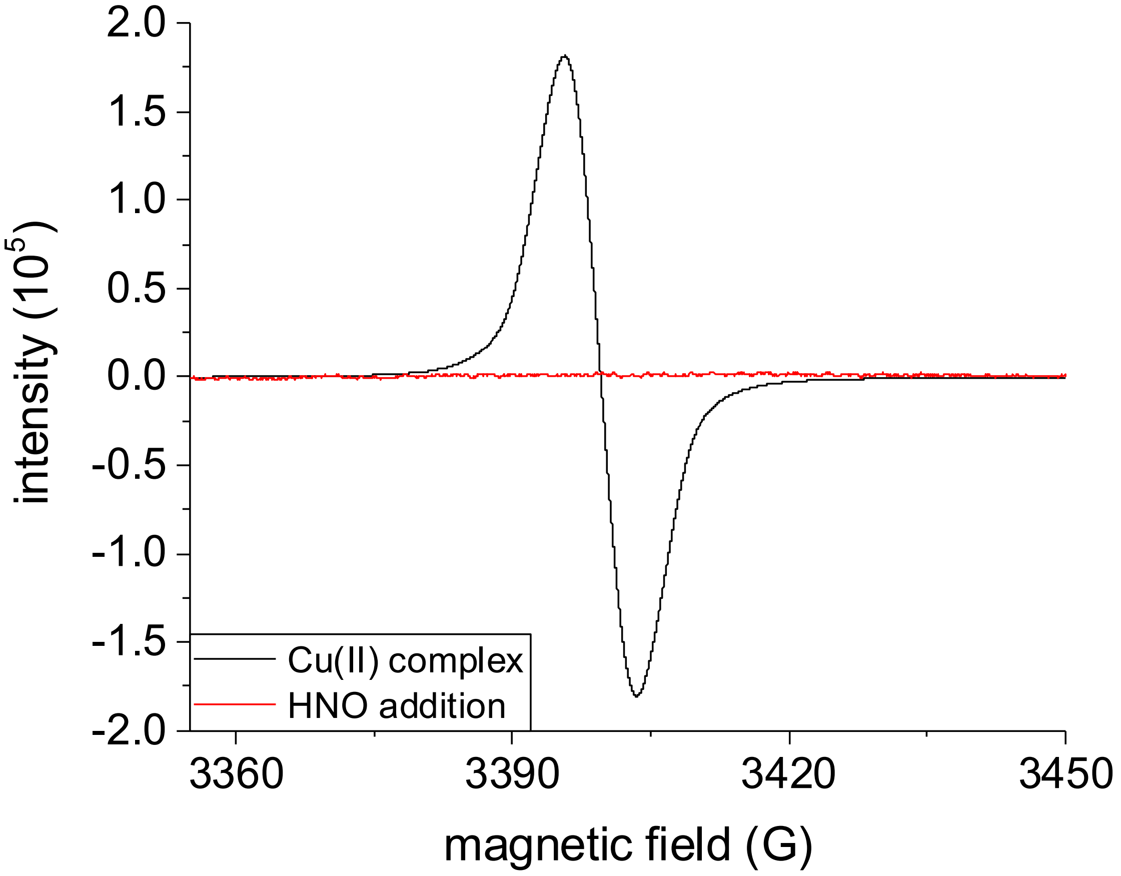

Figure S10. EPR spectra recorded at 298 K for 40 μM Cu(II)-AbTCA (black line) and with excess Angeli's salt (red line).

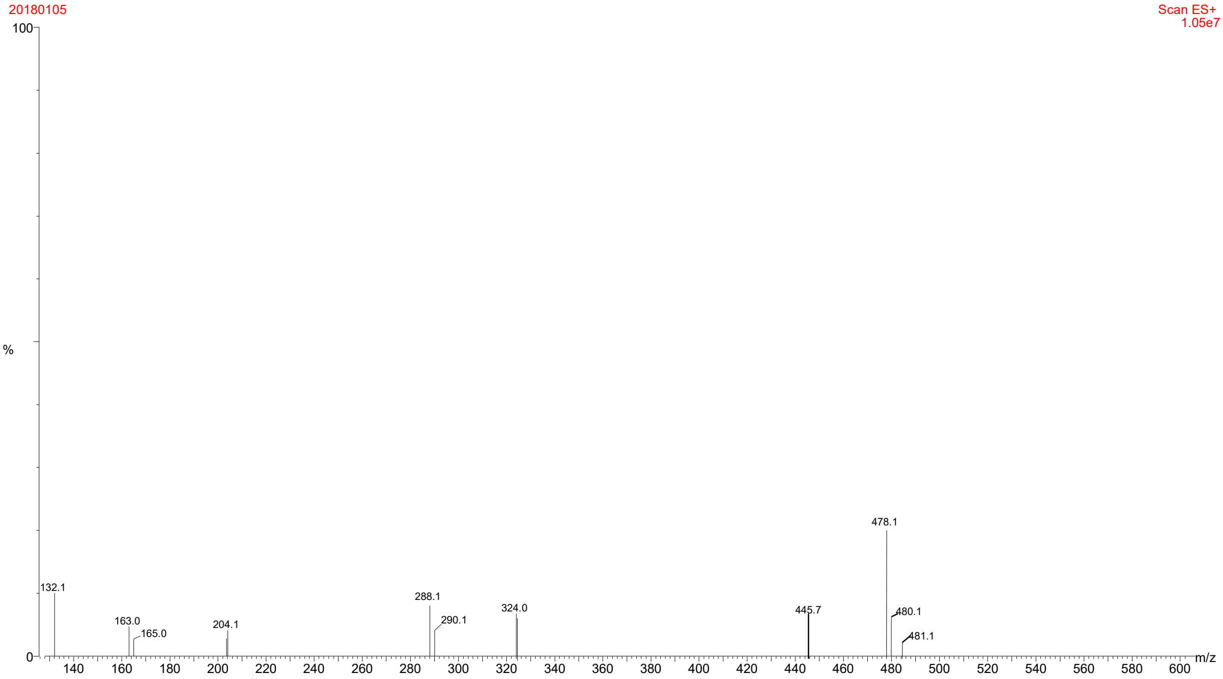

Figure S11. ESI-Mass spectrum of HNO treated Cu(II)-AbTCA

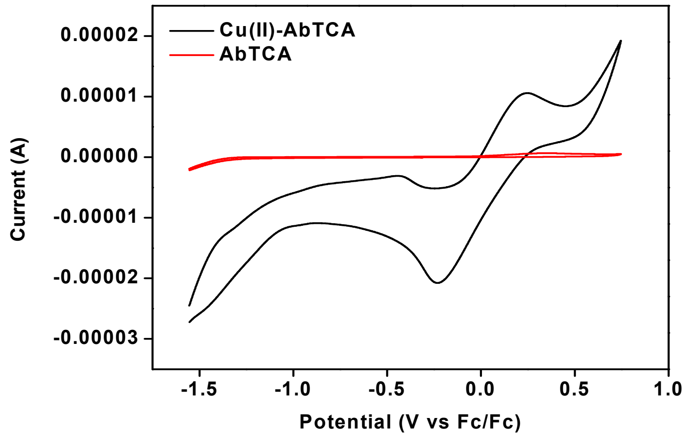

Figure S12. Cyclic voltammograms of AbTCA and Cu(II)-AbTCA

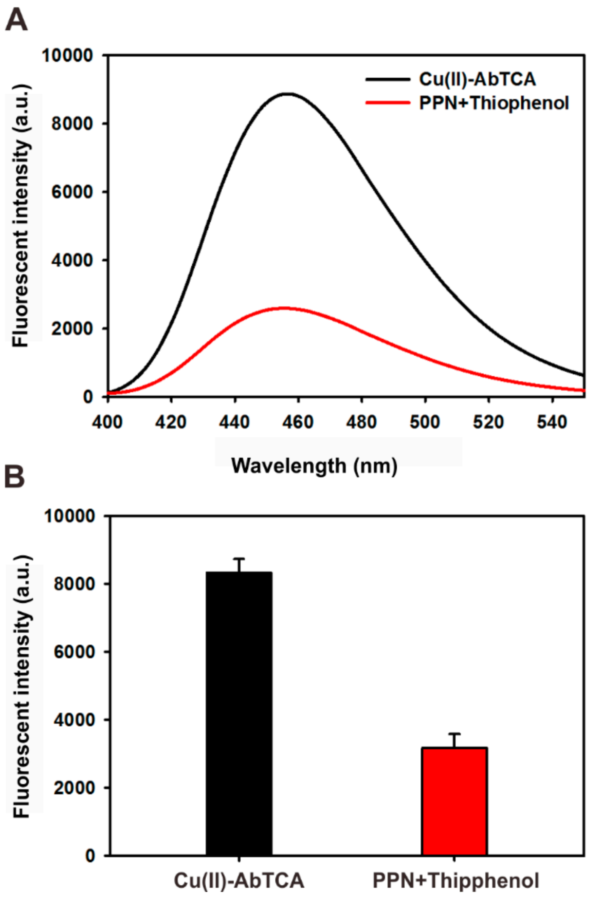

Figure S13. Detection of nitroxyl release from DNIC complex, $[PPN][Fe(NO)_4]$ and thiophenol using Cu(II)-AbTCA probe.

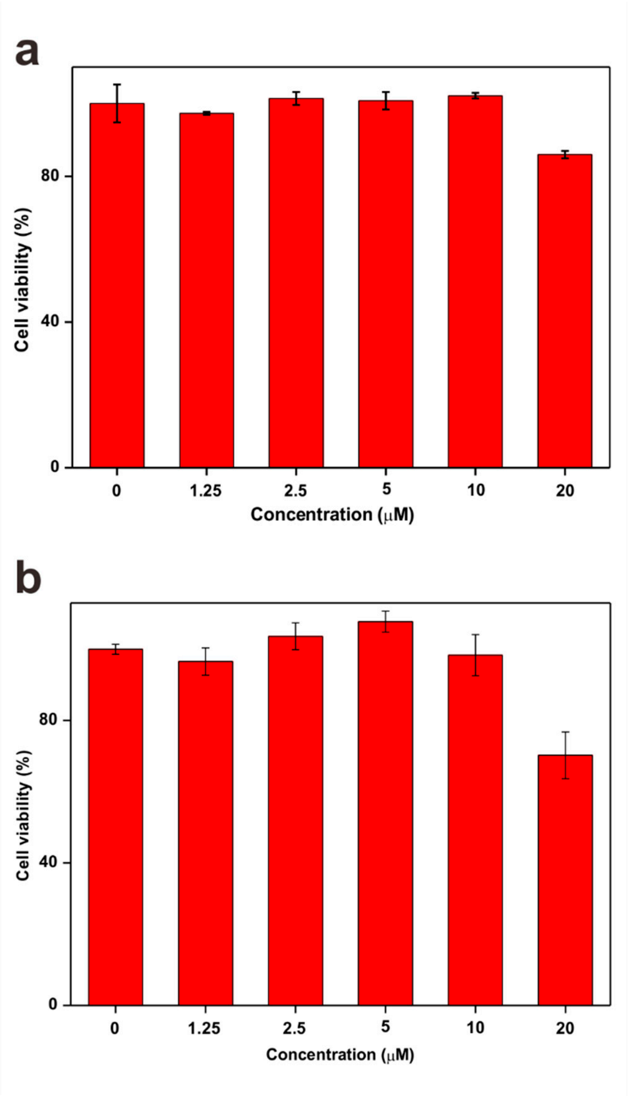

Figure S14. Evaluation of the potential cytotoxicity of Cu(II)-AbTCA to (A) EAHY-44926 cells; (B) RAW 264.7 cells.

References

1. Wrobel, A. T.; Johnstone, T. C.; Liang, A. D.; Lippard, S. J.; Fuentes, P. R. A Fast and Selective Near-Infrared Fluorescent Sensor for Multicolor Imaging of Biological Nitroxyl (HNO) *J. Am. Chem. Soc.* **2014**, 136, 4697–4705.
2. B. Valeur, *Molecular Fluorescence: Principles and Applications*, Wiley-VCH, 2001.
3. D. Oushiki, H. Kojima, T. Terai, M. Arita, K. Hanaoka, Y. Urano, T. Nagano, *J. Am. Chem. Soc.* **2010,** 132, 2795-2801.
4. F. Ma, M. Liu, Z. Wang and C. Zhang, *Chem. Commun.*, 2016, 52, 1218-1221.
